# Endocranial anatomy and phylogenetic position of the crocodylian *Eosuchus lerichei* from the late Paleocene of northwestern Europe and potential adaptations for transoceanic dispersal in gavialoids

**DOI:** 10.1002/ar.25569

**Published:** 2024-09-03

**Authors:** Paul M. J. Burke, Sophie A. Boerman, Gwendal Perrichon, Jeremy E. Martin, Thierry Smith, Johan Vellekoop, Philip D. Mannion

**Affiliations:** ^1^ Department of Earth Sciences University College London London UK; ^2^ Department of Earth and Environmental Sciences KU Leuven Leuven Belgium; ^3^ Directorate Earth and History of Life Royal Belgian Institute of Natural Sciences Brussels Belgium; ^4^ Université Claude Bernard Lyon 1 Villeurbanne France

**Keywords:** *Eosuchus*, gavialoid, neuroanatomy, oceanic dispersal, salt glands, thoracosaur

## Abstract

*Eosuchus lerichei* is a gavialoid crocodylian from late Paleocene marine deposits of northwestern Europe, known from a skull and lower jaws, as well as postcrania. Its sister taxon relationship with the approximately contemporaneous species *Eosuchus minor* from the east coast of the USA has been explained through transoceanic dispersal, indicating a capability for salt excretion that is absent in extant gavialoids. However, there is currently no anatomical evidence to support marine adaptation in extinct gavialoids. Furthermore, the placement of *Eosuchus* within Gavialoidea is labile, with some analyses supporting affinities with the Late Cretaceous to early Paleogene “thoracosaurs.” Here we present novel data on the internal and external anatomy of the skull of *E. lerichei* that enables a revised diagnosis, with 6 autapormorphies identified for the genus and 10 features that enable differentiation of the species from *Eosuchus minor*. Our phylogenetic analyses recover *Eosuchus* as an early diverging gavialid gavialoid that is not part of the “thoracosaur” group. In addition to thickened semi‐circular canal walls of the endosseous labyrinth and paratympanic sinus reduction, we identify potential osteological correlates for salt glands in the internal surface of the prefrontal and lacrimal bones of *E. lerichei*. These salt glands potentially provide anatomical evidence for the capability of transoceanic dispersal within *Eosuchus*, and we also identify them in the Late Cretaceous “thoracosaur” *Portugalosuchus*. Given that the earliest diverging and stratigraphically oldest gavialoids either have evidence for a nasal salt gland and/or have been recovered from marine deposits, this suggests the capacity for salt excretion might be ancestral for Gavialoidea. Mapping osteological and geological evidence for marine adaptation onto a phylogeny indicates that there was probably more than one independent loss/reduction in the capacity for salt excretion in gavialoids.

## 1 INTRODUCTION

The genus *Eosuchus* was erected by Dollo (1907) for the species *Eosuchus lerichei*, based on a well‐preserved skull and several postcranial elements from the upper Paleocene marine Hannut Formation that outcrops at Jeumont at the present‐day French‐Belgian border. The initial description was brief and was substantially improved upon by Delfino et al. (2005), who also provided photographs of the specimen for the first time. In parallel, Brochu (2006) revised the remains of specimens from upper Paleocene (and possibly lower Eocene) marine deposits of the Atlantic coast of the USA that had originally been described as *Gavialis minor* (Marsh, 1870) and later *Thecachampsoides minor* (Norell and Storrs, 1989), assigning them to a second species of *Eosuchus*, with the new combination *E*. *minor*. The two species of *Eosuchus* were recovered as sister taxa in the phylogenetic analyses of Delfino et al. (2005) and Brochu (2006), united by the following characteristics: (1) an enlarged foramen aereum of the quadrate; (2) an arrangement of dentary alveoli in couplets; (3) a long nasal process between the premaxillae; and (4) a “W”‐shaped basioccipital tuberosity. A sister taxon relationship between these two species has been consistently recovered in subsequent phylogenetic analyses (e.g., Brochu, 2007; Groh et al., 2020; Lee & Yates, 2018; Rio & Mannion, 2021; Salas‐Gismondi et al., 2022).

In most analyses, *Eosuchus* has been recovered as a gavialoid, typically placed as a close relative to a clade comprising *Argochampsa*, gryposuchines, and *Gavialis* (e.g., Delfino et al., 2005; Rio & Mannion, 2021). However, in some studies, *Eosuchus* clusters with “thoracosaurs,” a group of Late Cretaceous to early Paleogene marine eusuchians (e.g., *Eothoracosaurus*, *Portugalosuchus*, *Thoracosaurus*) that usually form a paraphyletic array (e.g., Brochu, 2004; Delfino et al., 2005; Mateus et al., 2019; Puértolas‐Pascual et al., 2023; Rio & Mannion, 2021), but might be monophyletic (Salas‐Gismondi et al., 2022; Burke et al., 2024). Within Gavialoidea, the usual placement of *Eosuchus* and “thoracosaurs” as more closely related to *Gavialis gangeticus* than to *Tomistoma schlegelii* is problematic, given that this would extend the proposed divergence time of these two extant species back tens of millions of years (Rio & Mannion, 2021), predating molecular‐based estimates which indicate a divergence time of 31–18 Ma (Oaks, 2011; Pan et al., 2021). A further complication is that “thoracosaurs”, including *Eosuchus*, are recovered as gavialoids in most analyses, but outside of Crocodylia in others (e.g., Darlim et al., 2022; Salas‐Gismondi et al., 2022).

The occurrence of *Eosuchus* in marine deposits on both sides of the North Atlantic Ocean has led several authors to explain this distribution through transoceanic dispersal within the lineage (see Brochu, 2006; Delfino et al., 2005). A similar scenario seems plausible for other contemporaneous species, with *Thoracosaurus isorhynchus* found in the Maastrichtian and early Paleocene of Europe (Koken, 1888; Troedsson, 1923), and *Thoracosaurus neocesariensis* found in the late Maastrichtian and early Paleocene of the eastern USA (Brochu, 2004). Moreover, it is generally assumed that the two extant gavialoid species, although currently restricted to freshwater environments, are derived from saltwater‐tolerant ancestors. This is based on the cosmopolitan distribution of fossil gavialoids, including their presence in marine deposits (e.g., Buffetaut, 1982; Brochu, 2003; Jouve et al., 2008; Martin et al., 2012; Piras et al., 2007; Rio & Mannion, 2021; Salas‐Gismondi et al., 2022), but also on the morphology of the buccal structure in living species, which potentially possess lingual salt glands (Taplin et al., 1985; Taplin and Grigg, 1989). Nevertheless, currently there is no clear anatomical information to assess whether *Eosuchus* or other putative “thoracosaurs” were capable of marine dispersal. One potential source of novel information comes from the evaluation of internal anatomical features of the skull, several of which have been shown to correspond to the paleocology of crocodylomorphs (e.g., Pierce et al., 2017; Schwab et al., 2020; Barrios et al., 2023; Ristevski, 2022; Cowgill et al., 2023; Perrichon et al., 2023a; Burke & Mannion, 2023).

Here, we present the first reconstruction of the internal anatomy of the skull of *E. lerichei*, specifically of the braincase and paratympanic sinus system, based on computed tomography (CT) scanning. We also provide an emended diagnosis of the species, including revised interpretations of some previously described anatomical features. We conduct phylogenetic analyses with updated scoring and make anatomical comparisons to “thoracosaurs” and other gavialoids and marine crocodyliforms. Furthermore, the newly acquired data on internal cranial anatomy enables us to make inferences about the paleoecology of *E. lerichei* and the likelihood of transoceanic dispersal in this lineage.

## 2 MATERIALS AND METHODS

### 2.1 Preservation and depositional environment

**FIGURE 1:**
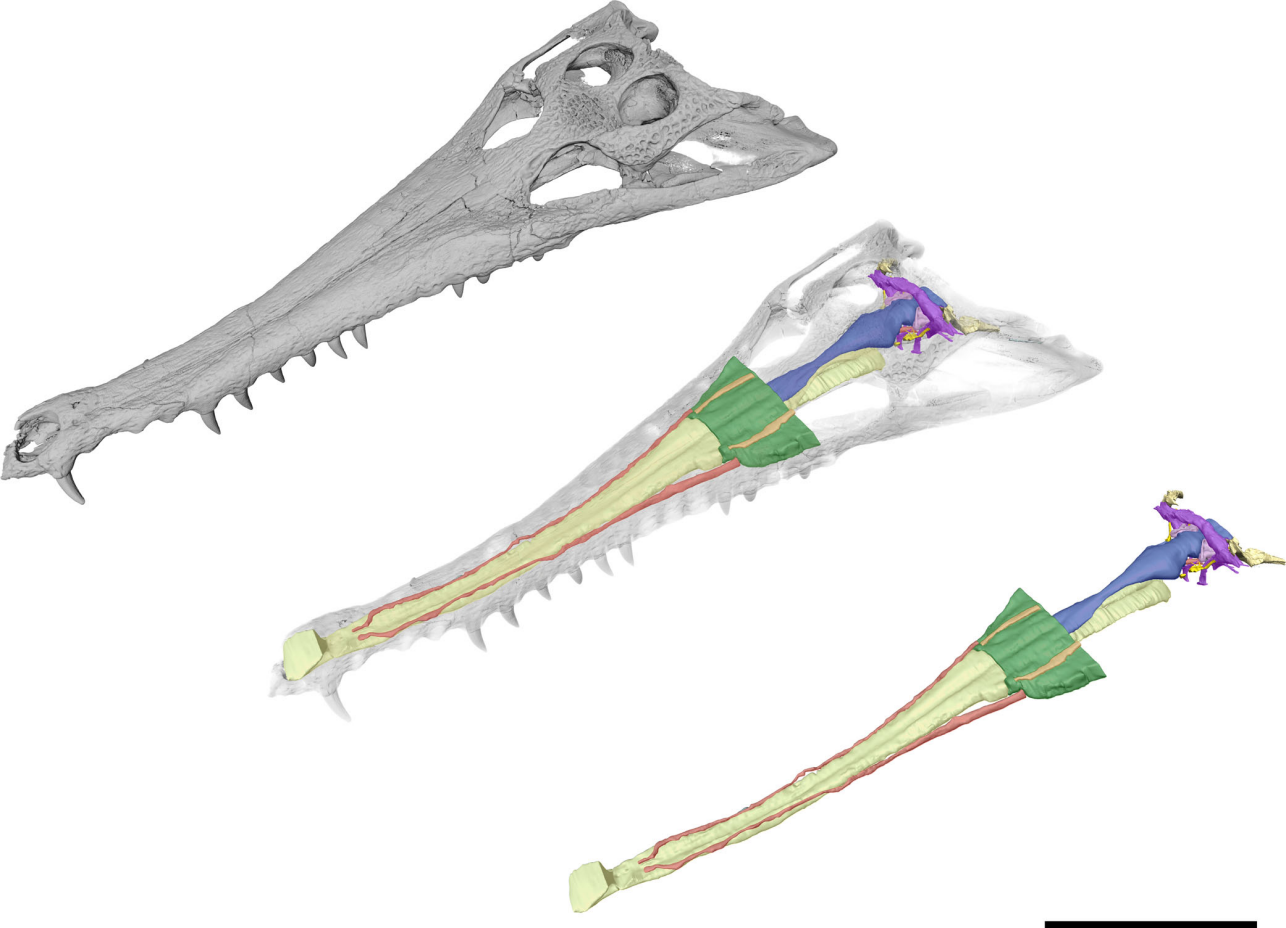
The skull and endocranial anatomy of *Eosuchus lerichei* (IRSNB R49) in left dorsolateral view. Yellow = nasal cavity, red = neurovascular canals, orange = nasolacrimal ducts, green = paranasal sinus, blue = brain endocast, pink = endosseous labyrinth, purple = paratympanic sinus, bright yellow = cranial nerves. Scale bar = 100 mm.

The endocranial structures of *E. lerichei* are described here based on the holotype specimen, Institut royal des Sciences naturalles de Belgique (IRSNB) R49, an almost complete cranium (Figure 1) associated with incomplete lower jaws, the axis, three postaxial cervical vertebrae, an isolated neural arch, three ribs, a fragmentary ulna and radius, a carpal element and 14 osteoderms, all pertaining to a single individual (Delfino et al., 2005). The skull was altered by dorsoventral deformation, which impacted the segmentation of the endocranial features (see Delfino et al., 2005). The specimen was discovered in January 1907 in the sand quarry of Martial Dusart and Son, in the township of Jeumont, northern France, at the French‐Belgian border; this quarry is located in the vicinity of nine other sand quarries, oriented in a south–north axis in the Erquelinnes area of western Belgium (Missiaen et al., 2013, fig. 1). It was collected in a plaster jacket in the field and then extracted from the sand matrix in the paleontological preparation laboratory of the IRSNB.

IRSNB R49, as well as several partial to almost complete skeletons of the choristoderan *Champsosaurus dolloi* and the cheloniid turtle *Erquelinnesia gosseleti*, were collected in a shallow marine non‐calcareous glauconitic sand unit of the Grandglise Sand Member, belonging to the upper Paleocene Hannut Formation (Lower Landenian in earlier literature; Dollo, 1907; Sigogneau−Russell & Heinzelin, 1979). This part of the Grandglise Sand Member corresponds to the mid‐Thanetian zone NP8 (De Coninck et al., 1981; Steurbaut, 1998).

### 2.2 CT‐scanning methodology

The cranium of IRSNB R49 was scanned at the X‐ray facility of IRSNB using a RX EasyTom micro‐CT scanner (RX Solutions, Chavanod, France; https://www.rx-solutions.com/). Scan parameters were set to 145 kV voltage, 448 μA intensity, 0.4 mm thick copper filter, 20,160 projections (stacking of seven scans of 2880 projections each), 4 h 52 min of exposure time, resulting in a voxel size of 76μm^3^. The raw CT slices were imported into the software ImageJ (Schneider et al., 2012), where the contrast was enhanced between bones, sediment infill and cavities. Image type was transformed from 16‐bit to 8‐bit and voxel size was reduced from 76 to 152 μm^3^ to reduce file size. A single file was then exported in RAW format and imported into the software Avizo v. 9.7 (FEI Visualization Science Group; https://www.thermofisher.com), in which internal cavities of the braincase and rostrum were segmented. The segmentation was performed manually, and 3D models of the internal structures were smoothed in Blender (Stichting Blender Foundation, Amsterdam) and rendered in Inkscape (Inkscape Project, 2020).

### 2.3 Phylogenetic dataset and analysis

We performed two phylogenetic analyses, adapting the matrix of Burke et al. (2024), which is an updated version of the matrix presented by Rio and Mannion (2021). The latter introduced many new characters focused on resolving the phylogenetic relationships of crocodylians and represents the first work to consistently retrieve a monophyletic Longirostres based on morphological data; thus, it is more consistent with results based on molecular datasets (e.g., Pan et al., 2021) than previous morphology‐based phylogenetic analyses. Several authors have subsequently used adaptations of this matrix (Boerman et al., 2023; Burke et al., 2024; Chabrol et al., 2024; Conedera et al., 2023; Puértolas‐Pascual et al., 2023).

The character scoring of *E. lerichei* was updated based on external observations and the internal morphology from the CT data. In addition, the scoring for character 86 (parietal, recess communicating with pneumatic system) was altered for several taxa in accordance with the findings of recent studies (Bona et al., 2015; Serrano‐Martínez et al., 2018, 2021, 2019; Puértolas‐Pascual et al., 2022, 2023; Perrichon et al., 2023a; Burke et al., 2024). An exhaustive list of changes made to the matrix of Burke et al. (2024) can be found in the Supplementary Material. The current version of the matrix presented here contains 151 taxa scored for 331 characters. However, based on our observations of the enlarged foramen on the posterior surface of the quadrate (see below under Section 4.4 Paratympanic sinuses), character 116 (quadrate, foramen aereum size) was found to be phylogenetically uninformative. In order to be consistent with the numbering of the characters compared to previous iterations of this matrix, we opted to retain this character in the matrix but to inactivate it during the analyses. Of the remaining 330 characters, the first 26 were treated as continuous, and 37 of the 304 discrete characters (characters 37, 47, 48, 58, 65, 72, 75, 78, 81, 87, 88, 102, 109, 110, 137, 142, 151, 162, 175, 181, 188, 210, 214, 216, 220, 221, 222, 224, 235, 243, 284, 293, 297, 308, 323, 324) were treated as ordered.

In TNT version 1.5 (Goloboff & Morales, 2023), a New Technology Search was first performed under equal weighting. All algorithms (Sect. Search, Ratchet, Drift and Tree fusing) were enabled, and the consensus tree was stabilized five times with a factor of 75. The default settings were used for all other options. After this initial search, a Traditional Search was performed using trees from RAM under the tree bisection and reconnection branch swapping algorithm. We then ran a second analysis under extended implied weighting, with a weighting factor (k) of 12, in which we applied the option to “downweight characters with missing entries faster,” with the other options left as the default settings.

## 3 SYSTEMATIC PALEONTOLOGY

Crocodylia Gmelin, 1789

Gavialoidea Hay, 1930

*Eosuchus* Dollo, 1907

**Emended diagnosis:** (1) anterior process of the nasals intruding in between the premaxillae until the level of the second maxillary alveolus; (2) dentary alveoli seven through 12 arranged in couplets (see Delfino et al., 2005: fig. 5)*; (3) anteriormost part of the postorbital dorsal surface ventrolaterally inclined instead of horizontal; (4) W‐shaped tuberosity on the ventral surface of the basioccipital (see Brochu, 2006: fig. 15)*; (5) ventrolaterally directed crest extending from the ventral surface of the quadrate onto the lateral surface of the braincase wall (Brochu, 2006: fig. 15)*; and (6) large “blind” depression on the dorsal quadrate surface, with a diameter of at least half the dorsoventral height of the medial hemicondyle*. The latter feature was previously erroneously interpreted as the quadrate foramen aereum (Delfino et al., 2005; Brochu, 2006; see below). Apomorphic features of the genus are indicated with an asterisk.

**Type species**—*E. lerichei* Dollo, 1907

**Species assigned**: *E. lerichei* Dollo, 1907 and *Eosuchus minor* (Marsh, 1870)

*E. lerichei* Dollo, 1907

**Holotype**—IRSNB R49, an almost complete cranium associated with incomplete lower jaws, the axis, three cervical vertebrae, an isolated neural arch, three ribs, a fragmentary ulna and radius, a carpal element and 14 osteoderms, all belonging to a single individual (see Delfino et al., 2005).

**Type locality and horizon**—Jeumont, north of Maubeuge, Nord Department, France. The locality, a few meters from the French‐Belgian border, is part of the well‐known fossiliferous area of Erquelinnes, Belgium. Grandglise Member, Hannut Formation, Thanetian, Upper Paleocene.

**FIGURE 2:**
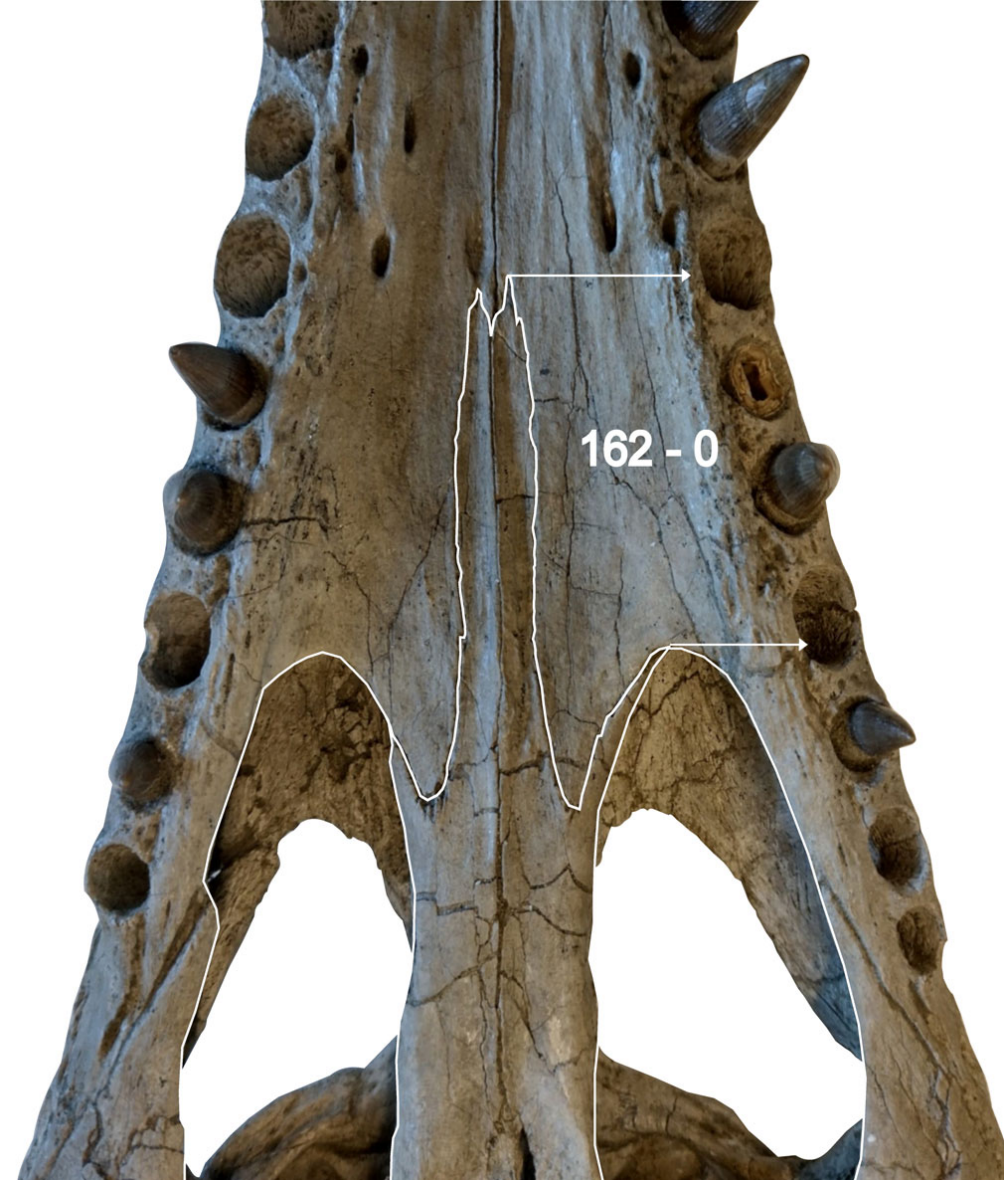
Palate of *Eosuchus lerichei* (IRSNB R49) highlighting an autapomorphy in which the anterior process of the palatine extends further than two full maxillary alveoli from the anterior margin of the suborbital fenestra.

**FIGURE 3:**
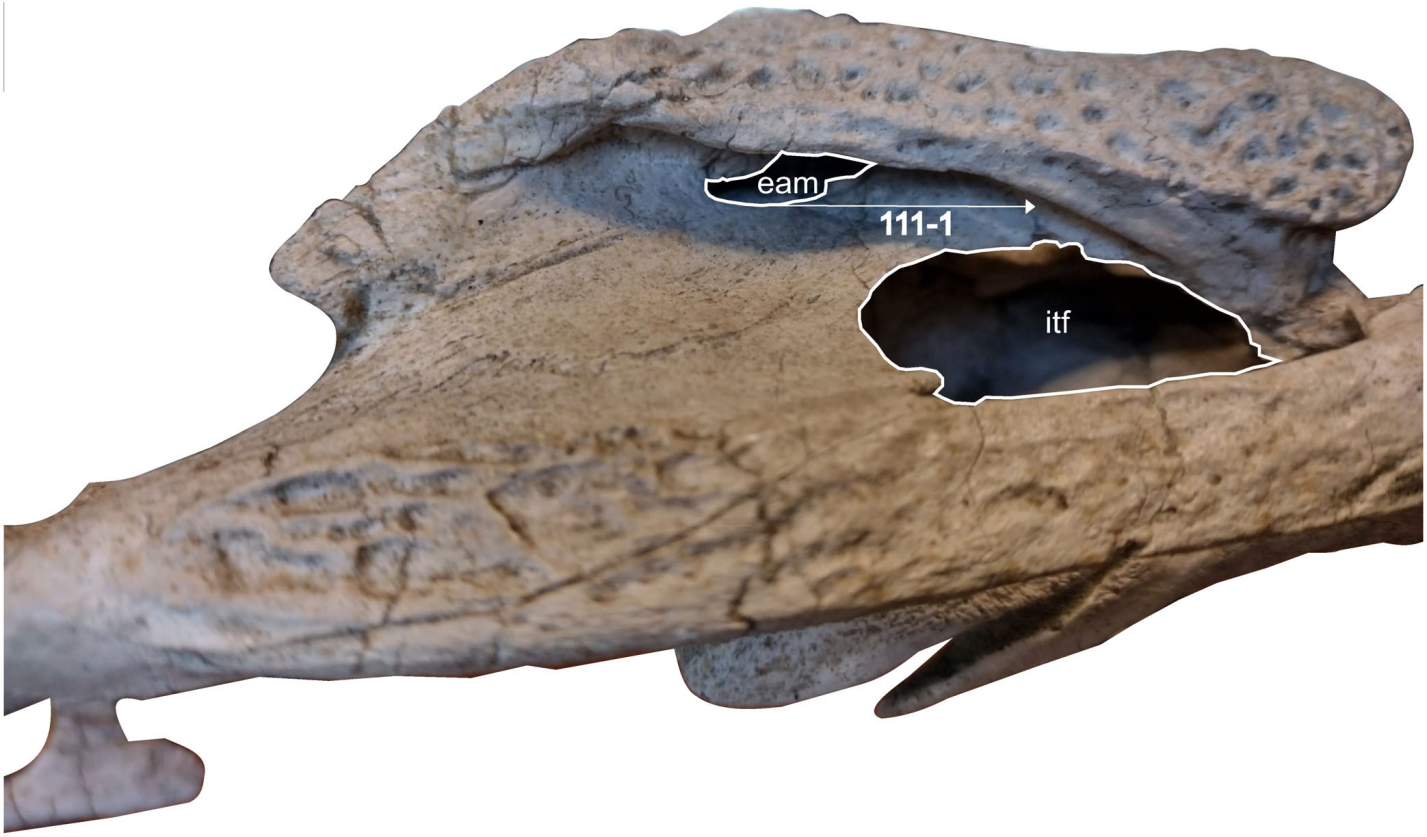
Temporal and auditory region of *Eosuchus lerichei* (IRSNB R49) in left dorsolateral view, highlighting an autapomorphy wherein the ventral margin of the external auditory meatus is positioned dorsally to the dorsal margin of the infratemporal fenestra.

**FIGURE 4:**
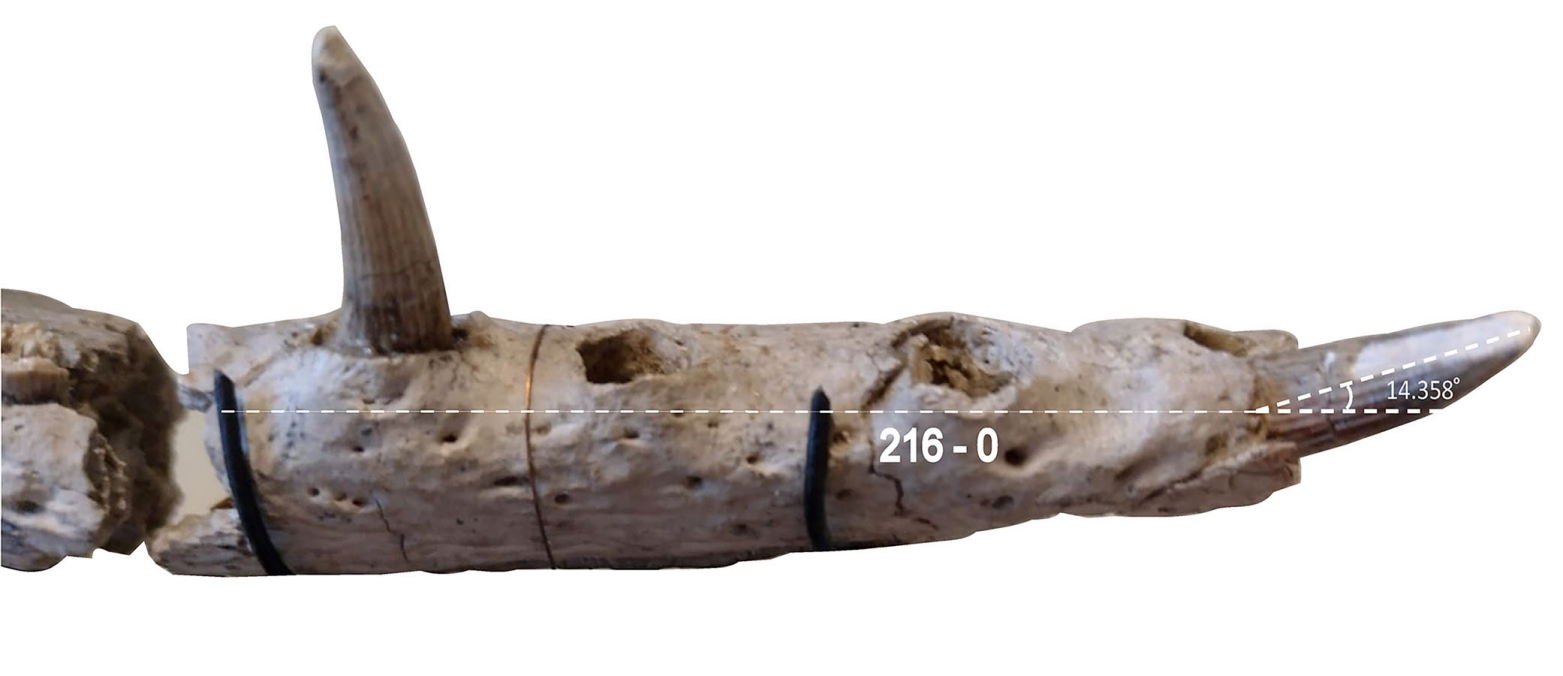
Anteriormost end of right dentary of *Eosuchus lerichei* (IRSNB R49) in left lateral view, highlighting an autapomorphy wherein the anteriormost dentary teeth are strongly procumbent, protruding from the dentary at a sub‐horizontal angle.

**Emended diagnosis:** *E. lerichei* differs from *E*. *minor* by: (1) the premaxilla extending posteriorly on the palate to the level of the second maxillary alveolus, as opposed to the first in *E*. *minor* (see Delfino et al., 2005: fig. 3 and Brochu, 2006: fig. 9); (2) the anterior process of the frontal forming a broad sutural contact with the nasals, which is unique to *E*. *lerichei* among crocodylians, whereas it forms an acute, “v”‐shape in E. minor (see Delfino et al., 2005: fig. 2 and Brochu, 2006: fig. 2); (3) the palatine anterior process extending to the level of more than two full alveoli anteriorly to the anterior margin of the suborbital fenestra (Figure 2), whereas the process extends to the level of two alveoli in *E*. *minor*; (4) the intersection of the maxilla‐palatine suture occurring at the anterior corner of the suborbital fenestra (Figure 2), but at the anteromedial margin of the suborbital fenestra in *E*. *minor*; (5) the ventral margin of the auditory meatus positioned dorsal to the level of the dorsal margin of the infratemporal fenestra (Figure 3), whereas it is positioned ventrally in *E*. *minor*; (6) the quadratojugal not reaching the dorsal margin of the infratemporal fenestra, whereas it does in *E*. *minor*; (7) the anteriormost dentary teeth strongly procumbent, protruding from the dentary at as sub‐horizontal angle, whereas they protrude at an angle greater than 60° in *E*. *minor* (Figure 4); (8) the symphysis of the lower jaw extending posteriorly to the level of the sixteenth dentary alveolus (see Delfino et al., 2005: fig. 5), whereas they only extend between the ninth and twelfth alveoli in *E*. *minor*; (9) the dorsoventrally thin, rod‐like axis neural spine (see Delfino et al., 2005: fig. 6), which is dorsoventrally thick in *E*. *minor*; and (10) the unforked axial hypapophysis, which is forked in *E*. *minor*.

## 4 DESCRIPTION AND COMPARISONS

### 4.1 Brain endocast

**FIGURE 5:**
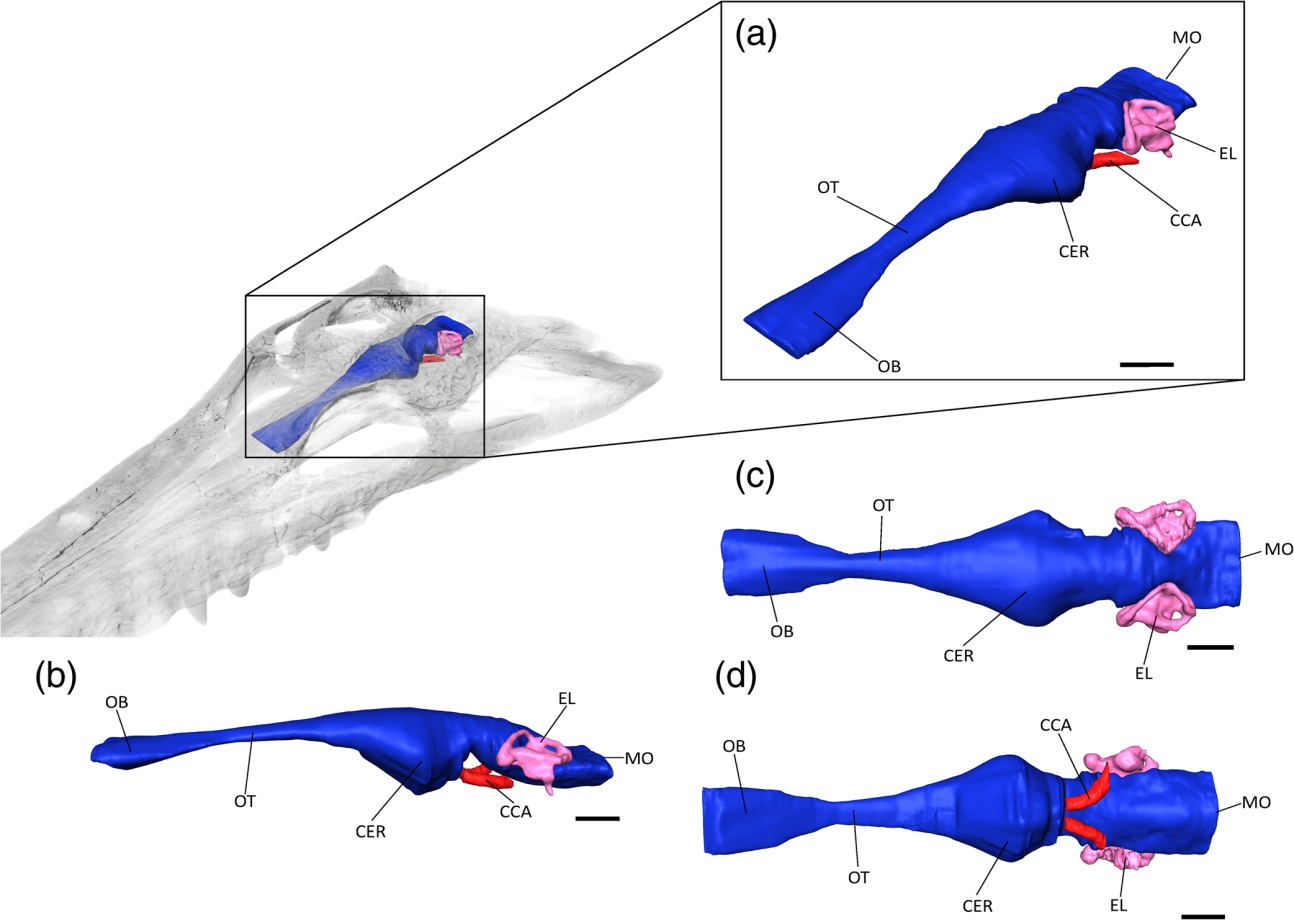
The brain endocast of *Eosuchus lerichei* (IRSNB R49) in (a) anterior oblique, (b) lateral view, (c) dorsal, and (d) ventral view. CER, cerebrum; CCA, cerebral carotid arteries; MO, medulla oblongata; OB, olfactory bulb; OT, olfactory tract; PIT, pituitary fossa. Scale bars = 10 mm.

**FIGURE 6:**
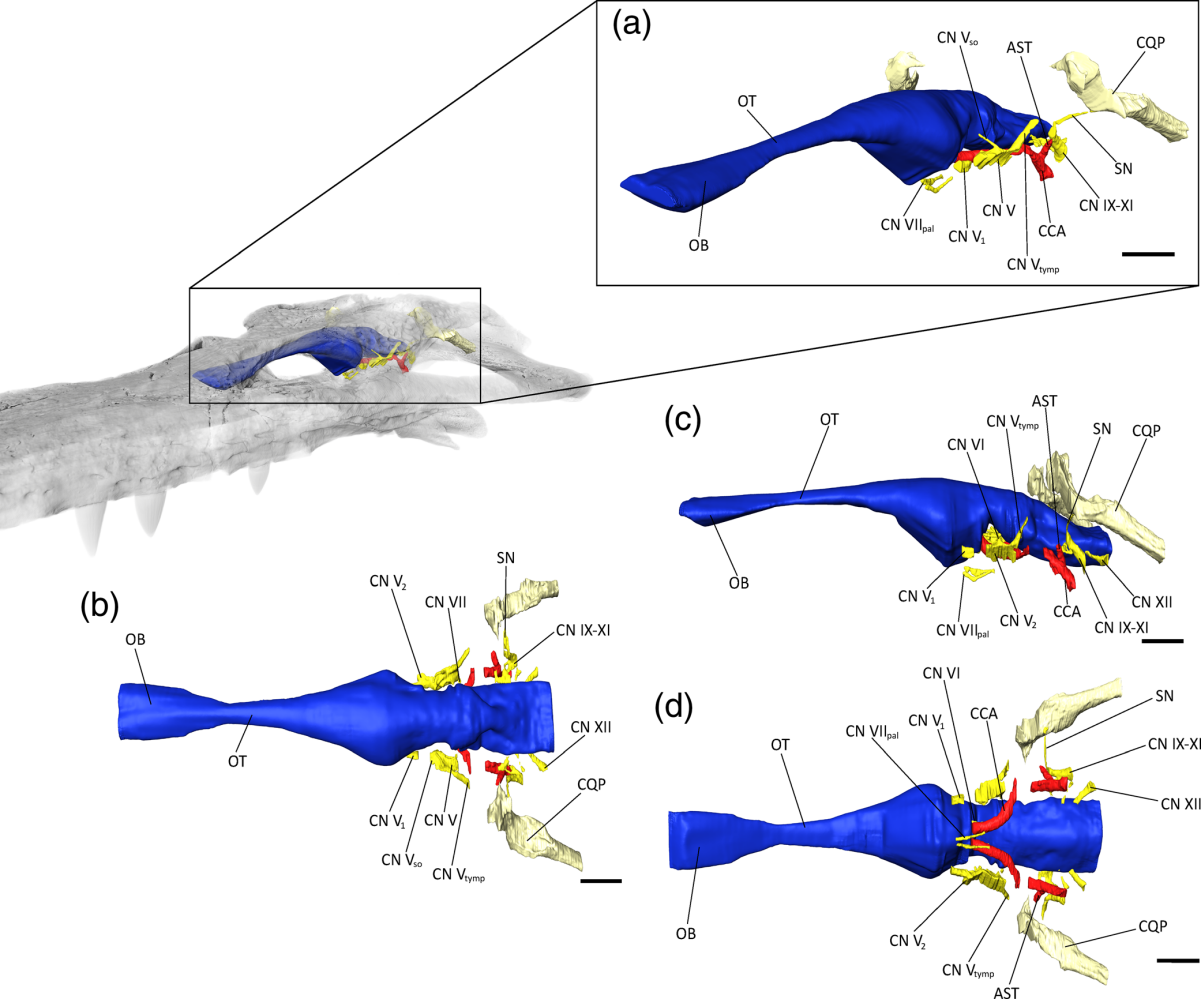
Brain endocast and neurovascular system of *Eosuchus lerichei* (IRSNB R49) in (a) anterior oblique, (b) dorsal, (c) lateral, and (d) ventral view. AST, arterial branch linking the stapedial artery to the cerebral carotid artery; CCA, cerebral carotid arteries; CN V, trigeminal ganglion; CN V_1_, ophthalmic branch of CN V; CN V_2_, mandibular branch of CN V; CN V_so_, supraorbital branch of CN V; CN V_tymp_, tympanic branch of CN V; CN VI, abducens nerve; CN VII, facial nerve; CN VII_pal_, palatine branch of CN VII; CN IX‐XI, common canal for the posterior cranial nerves; CQP, cranioquadrate passage; OB, olfactory bulb; OT, olfactory tract; SN, spinal accessory nerve. Scale bars = 10 mm.

**FIGURE 7:**
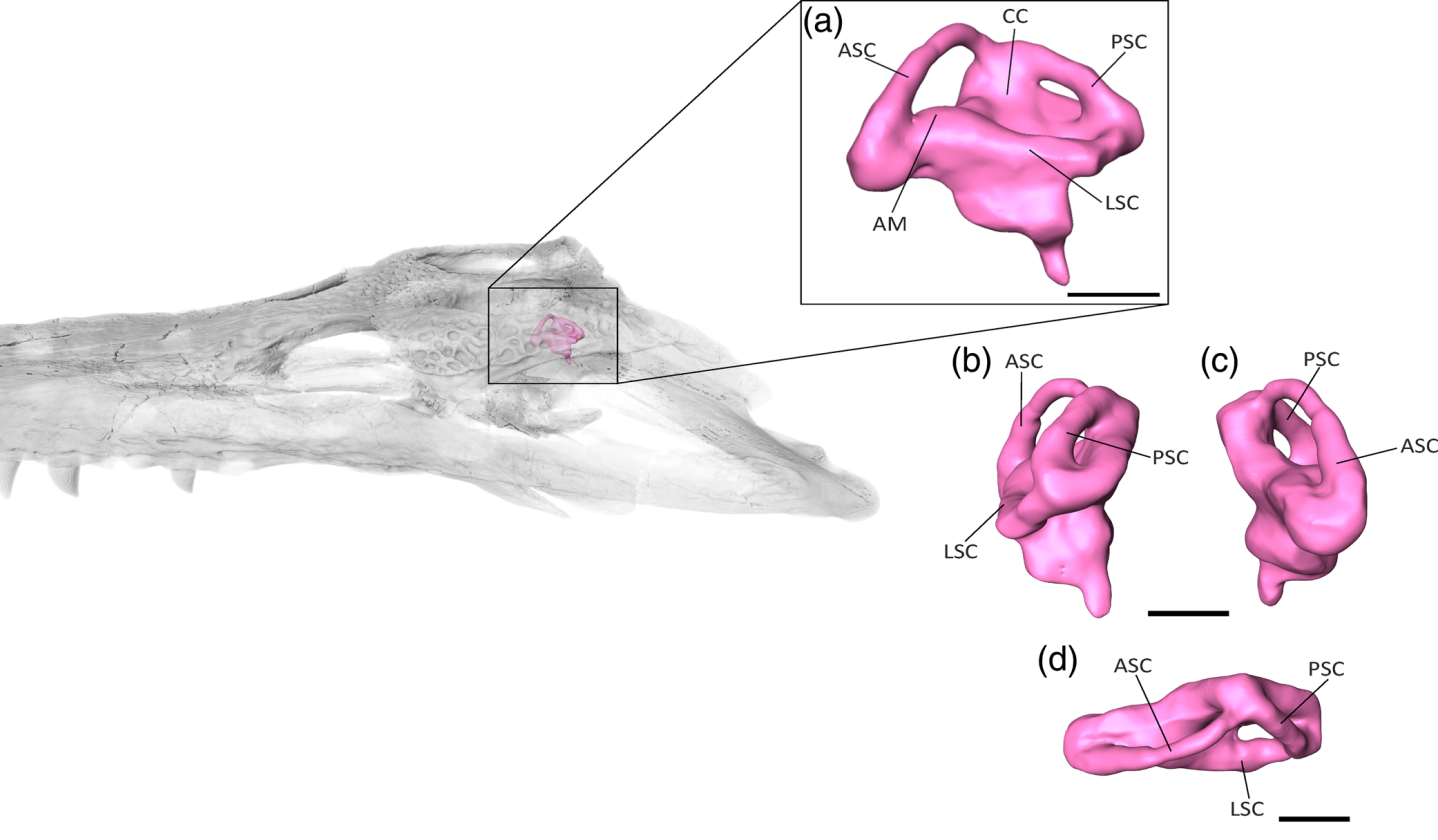
The endosseous labyrinth of *Eosuchus lerichei* (IRSNB R49) in (a) lateral, (b) right lateral, (c) left lateral and (d) ventral view. AM, ampullae; ASC, anterior semicircular canal; CC, common crux; LSC, lateral semicircular canal; PSC, posterior semicircular canal. Scale bars = 10 mm.

The encephalic endocasts of adult, slender longirostrine crocodylians are typically relatively straight in lateral view, showing little curvature (Burke & Mannion, 2023; Edinger, 1938; Hopson & Gans, 1979). *E. lerichei* reflects this, showing a slight curvature in the olfactory tract, but overall remaining straight and subhorizontal in lateral view, as demonstrated by the cephalic and pontine flexure angles of the endocast (Supplemental Material Table 1), which are similar to the morphology in *Gavialis gangeticus* (Figure 5). At its most anterior point, the encephalic endocast of *E. lerichei* is characterized by an olfactory bulb, the region of which is triangular in outline, that decreases in mediolateral width posteriorly and is connected to the cerebrum by the olfactory tract (Figures 5, 6, 7). In some extant species of Crocodylia, including *Gavialis gangeticus*, there is a lack of osteological division between the olfactory tract and bulb (Burke & Mannion, 2023; Pierce et al., 2017); this is also the case in *E. lerichei*, resulting in a gradual transition between the bulb and tract (Figures 5, 6, 7).

The cerebrum of *E. lerichei* is the most laterally expansive part of the encephalic endocast, with a width to that of the skull ratio consistent with other slender longirostrine crocodyliforms (Burke & Mannion, 2023; Erb & Turner, 2021; Pierce et al., 2017). In *E. lerichei*, the greatest expansion of the cerebrum occurs at its most posterior point, narrowing anteriorly toward the olfactory tract (Figure 5). This is similar to the morphology seen in other gavialoids, including *Gavialis gangeticus* and the extinct species “*Tomistoma*” *dowsoni* and *Portugalosuchus azenhae*, as well as several other crocodyliforms (e.g., Burke & Mannion, 2023; Colbert et al., 1946; Edinger, 1938; Hopson & Gans, 1979; Kley et al., 2010; Pierce et al., 2017; Puértolas‐Pascual et al., 2023). The pituitary is situated posteroventral to the cerebrum in *E. lerichei*, but it is difficult to distinguish from the rest of the encephalic endocast (Figure 5). Nevertheless, the channels that house the cerebral carotid arteries, which extend posterolaterally from the pituitary, are visible in *E. lerichei*. As in other crocodylians, they also curve dorsolaterally at their most posterior points, indicating the presence of the pituitary (Hopson & Gans, 1979; Witmer & Ridgely, 2008; Dufeau & Witmer, 2015; Pierce et al., 2017; Burke & Mannion, 2023).

### 4.2 Cranial nerves and vascular canals

The ventral parts of the forebrain and midbrain, as well as the medial side of the otic capsule, are poorly preserved as a consequence of the dorsoventral crushing of the laterosphenoids, prootics, and otoccipitals. Consequently, the cranial nerves II (optic), III (occulomotor), IV (trochlear), and VIII (vestibulocochlear) could not be reconstructed. The rest of the cranial nerves are better preserved on the left side of the skull (Figure 6) and are largely similar to those of extant crocodylians (Kuzmin et al., 2021). Cranial nerve I connects the anterior tip of the olfactory bulb with the nasal cavity (Lessner & Holliday, 2022), and as such could not be reconstructed.

The trigeminal nerve complex (CN V) is the largest neural structure connected to the brain, as in all crocodylians (George & Holliday, 2013). The trigeminal ganglion connects directly to the brain and is bounded by the laterosphenoid anteriorly and by the prootic posteriorly, with a possible contribution of the basisphenoid medioventrally. Several neural branches are rooted in the trigeminal ganglion, but only two of them are completely preserved on each side of the brain: the tympanic branch (CN V_tymp_), which is directed posterodorsally through the prootic and the quadrate, and the supraorbital branch (CN V_so_), which is directed anterodorsally through the laterosphenoid, re‐entering the forebrain cavity. From the left trigeminal ganglion, a large groove runs anteroposteriorly along laterosphenoid lateral surface and likely corresponds to the course of the ophthalmic (CN V_1_) and mandibular (CN V_2_) branches (Figure 6).

Posterolateral to the pituitary fossa, the abducens nerve (CN VI) is enclosed by the basisphenoid. This cranial nerve originates at the anteroventral part of the hindbrain and exits the basisphenoid lateral to the pituitary fossa, showing a slight ventral curvature (Lessner & Holliday, 2022).

The facial nerve (CN VII) joins the brain through a small foramen located anteroventral to the endosseous labyrinth. Its medial part is short upon entering the pharyngotympanic cavity. Only the ventralmost part of the palatine branches of the facial nerve (CN VII_pal_) could be reconstructed: they connect to the anterior part of the paratympanic sinuses and exit the braincase close to the basisphenoid rostrum.

The common canal for the posterior cranial nerves (CN IX–XI) hosts the glossopharyngeal (CN IX), vagus (CN X), spinal accessory (CN XI), and sympathetic nerves (SN) (Figure 6). On the otoccipital face of the skull, this canal is visible as a large foramen dorsal to the cerebral carotid artery. Inside the otoccipital, this common canal split in three. The canal for the sympathetic nerve separates from the cranial nerves IX‐XI at the level of the otic bulla and ascends dorsolaterally into the otoccipital. Its course is marked by a groove which joins the cranioquadrate passage dorsally. The two small canals for cranial nerves IX and X‐XI are directed anteromedially and join the brain endocast through the metotic foramen, ventral to the otic bulla (Figure 6).

The hypoglossal nerves (CN XII) connect laterally to the medulla oblongata, posterior to the metotic foramen. The anterior one (CN XII_1_) is thin and exits the otoccipital laterally, whereas the posterior one (CN XII_2_) is larger and directed posterolaterally (Figure 6).

The temporal canal is closed on both sides of the skull. The cranioquadrate passage is fully enclosed by bone and follows the quadrate‐otoccipital suture posteroventrally from the back of the meatal chamber. It slightly tapers posteriorly and exits the skull via a groove that separates the quadrate and the otoccipital. Anteriorly, the dorsoventral flattening of the skull has caused the squamosal to crush the posterior part of the meatal chamber.

The paired cerebral carotid arteries are not preserved in the prootic. At the level of the metotic foramen, a large secondary canal separates from the cerebral carotid artery (Figure 6): it corresponds to the arterial branch, linking the cerebral carotid arteries to the stapedial artery dorsally (sensu Kuzmin et al., 2021; Porter et al., 2016). It is directed dorsolaterally inside the otoccipital and enters the posterior wall of the pharyngotympanic cavity. This arterial branch is unusually large compared to that of extant species, in which it is generally a very thin canal (ø < 1 mm) located in the extracapsular buttress of the otoccipital (Kuzmin et al., 2021).

### 4.3 Endosseous labyrinth

**FIGURE 8:**
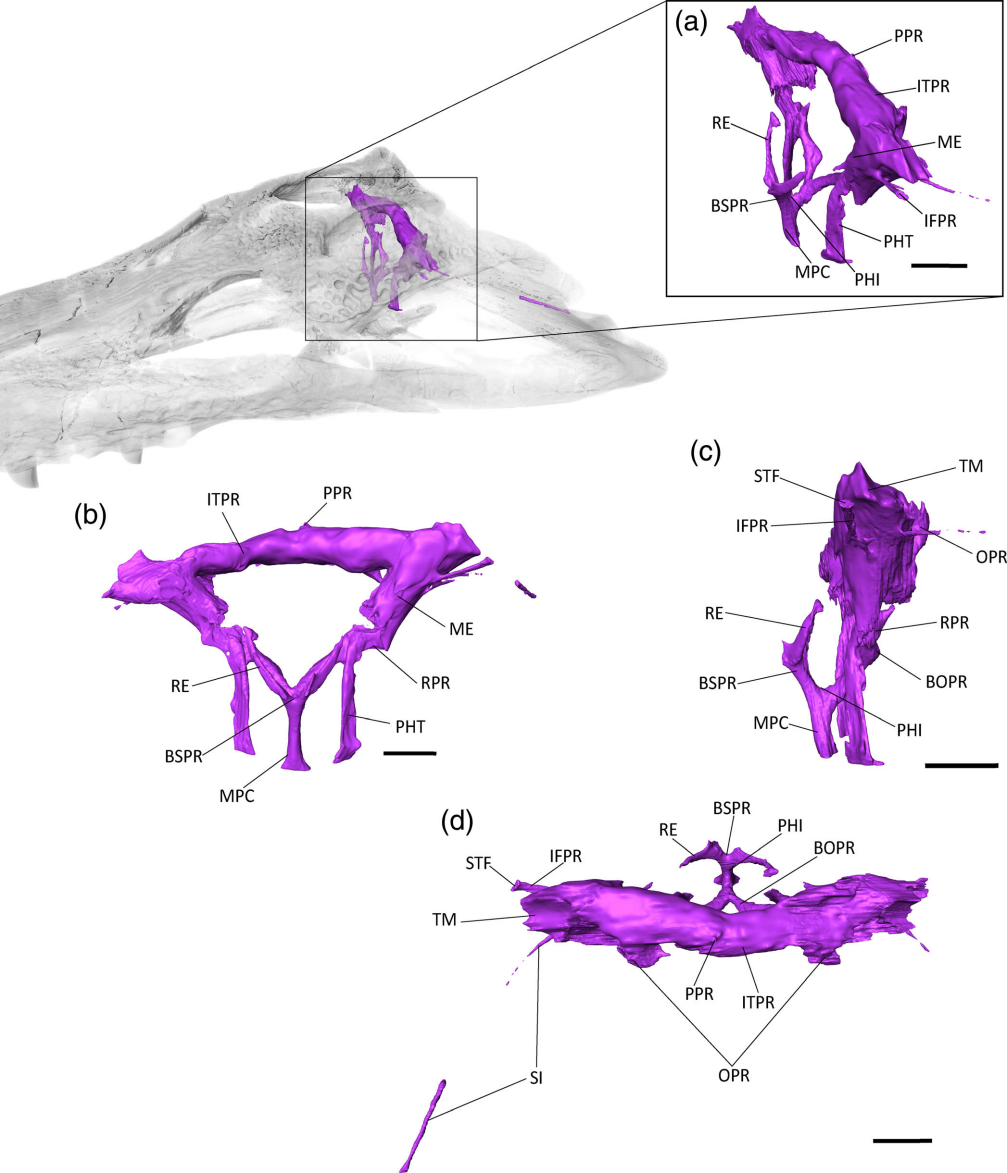
The paratympanic sinus system of *Eosuchus lerichei* (IRSNB R49) in (a) anterior oblique, (b) anterior, (c) lateral and (d) dorsal view. BOPR, basioccipital pneumatic recess; BSPR, basiosphenoid pneumatic recess; IFPR, infundibular pneumatic recess; ITPR, intertympanic pneumatic recess; ME, middle ear; MPC, median pharyngeal canal; OPR, otoccipital pneumatic recess; PHI, pharyngeal intersection; PHT, pharyngotympanic tubes; PP, posterior pre‐parietal process; RE, *recessus epitubaricus*; RPR, rhomboidal recess; SI, siphonium; STF, subtympanic foramen; TM, tympanic membrane. Scale bars = 10 mm.

As in most archosaurs (Brusatte et al., 2016), the anterior semi‐circular canal is larger than the posterior semi‐circular canal in *E. lerichei* (Figure 7). The walls of the semi‐circular canals are thicker than those of other gavialoids, including *Gavialis gangeticus* and *Tomistoma schlegelii*, as well as extinct *Gryposuchus neogaeus*, *Gunggamarandu maunala* and “*Tomistoma*” *dowsoni* (Bona et al., 2015; Burke & Mannion, 2023; Ristevski et al., 2021). In general, the overall shape appears more similar to gavialoids than to other crocodylians (Burke & Mannion, 2023). The cochlear duct is barely preserved. The lateral semi‐circular canal descends posterolaterally, differing from other gavialoids in which it is orientated anteroposteriorly (Figure 8; Burke & Mannion, 2023). The ampullae of the semicircular canals are large, and highly distinguishable from the canals (Figure 7).

### 4.4 Paratympanic sinuses

**FIGURE 9:**
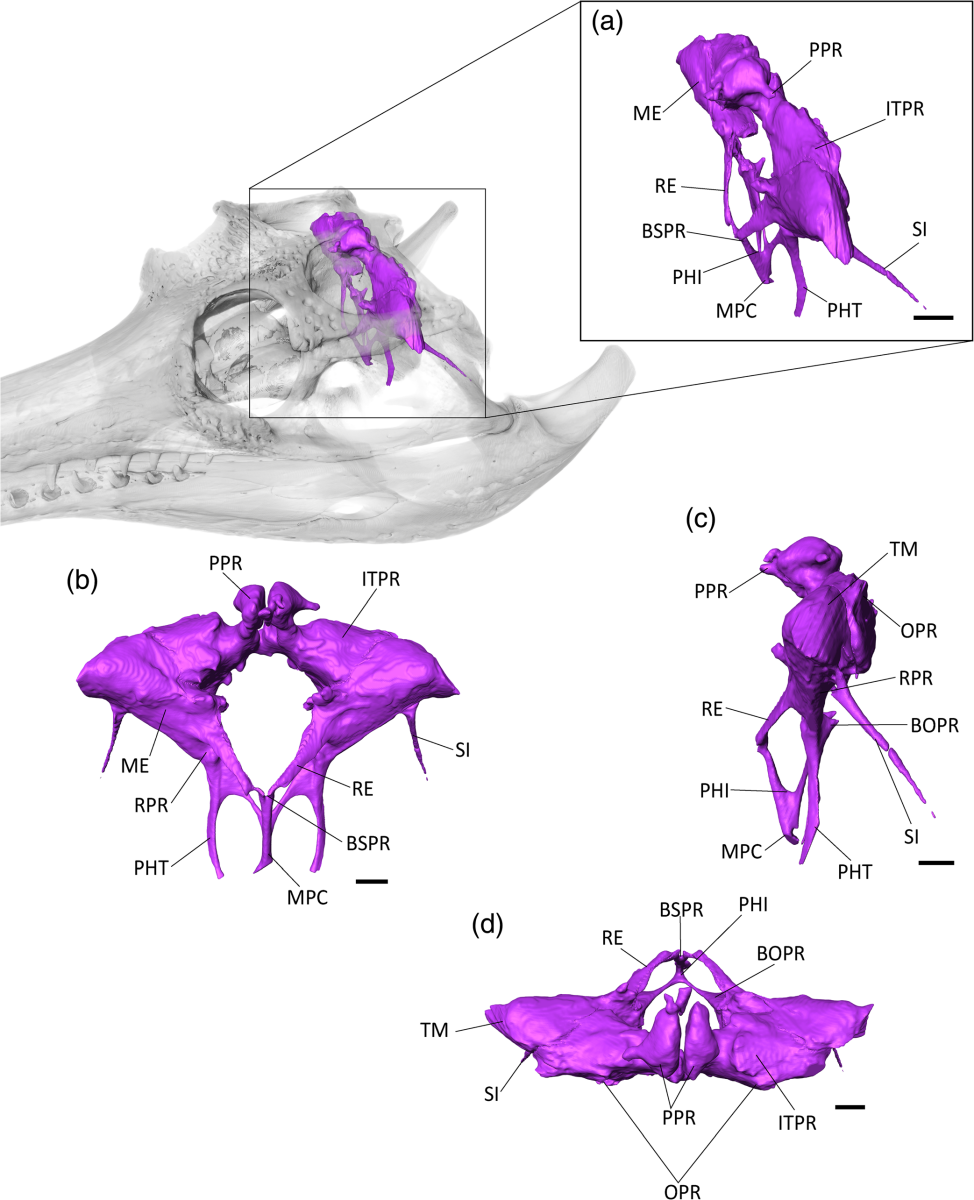
The paratympanic sinus system of *Gavialis gangeticus* (UF 118998) in (a) anterior oblique, (b) anterior, (c) lateral, and (d) dorsal view. BOPR, basioccipital pneumatic recess; BSPR, basiosphenoid pneumatic recess; ITPR, intertympanic pneumatic recess; ME, middle ear; MPC, median pharyngeal canal; OPR, otoccipital pneumatic recess; PHI, pharyngeal intersection; PHT, pharyngotympanic tubes; PP, posterior pre‐parietal process; RE, *recessus epitubaricus*; RPR, rhomboidal recess; SI, siphonium; TM, tympanic membrane. Scale bars = 10 mm.

**FIGURE 10:**
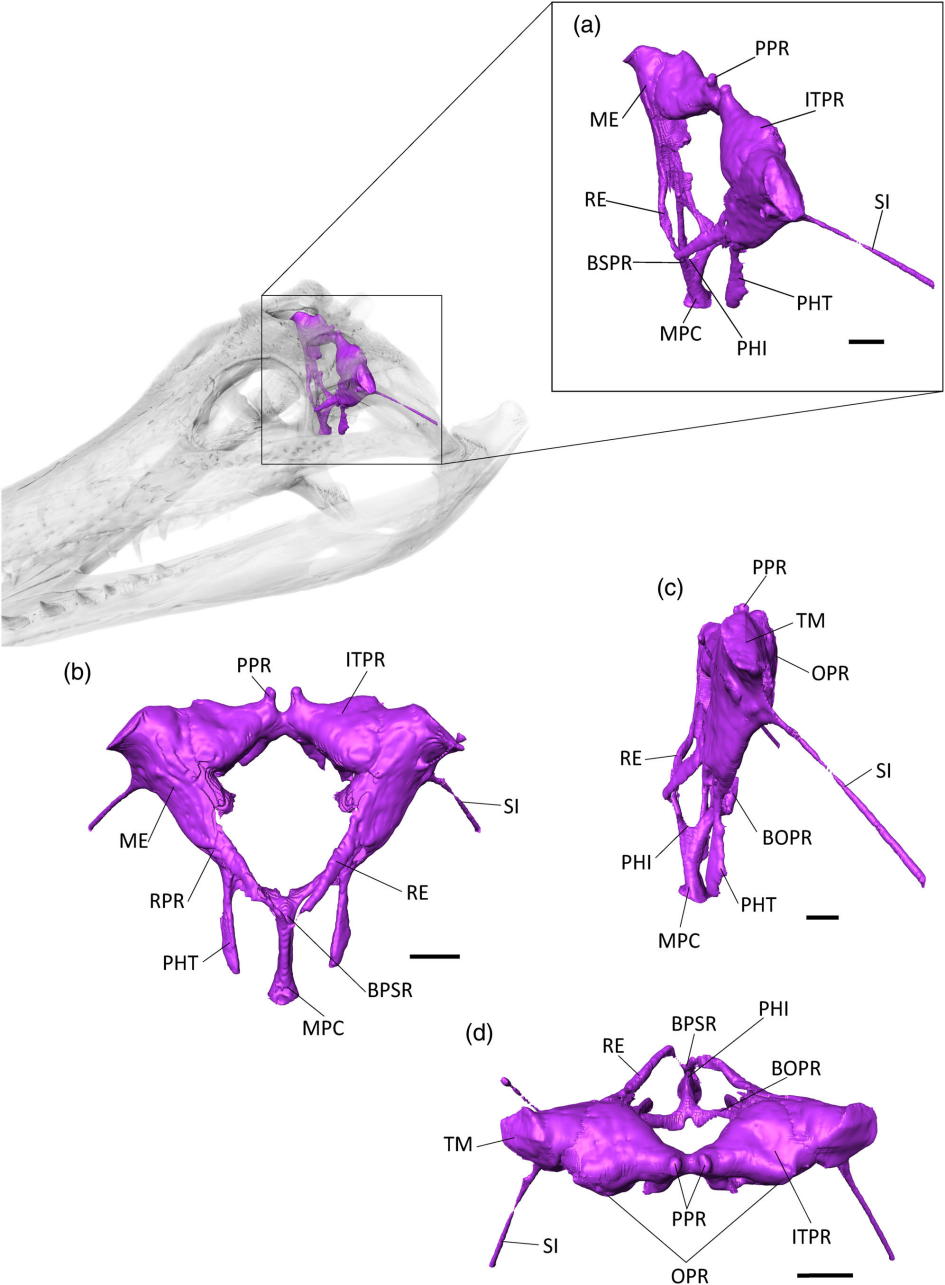
The paratympanic sinus system of *Tomistoma schlegelii* (TMM M6342) in (a) anterior oblique, (b) anterior, (c) lateral, and (d) dorsal view. BOPR, basioccipital pneumatic recess; BSPR, basiosphenoid pneumatic recess; IFPR, infundibular pneumatic recess; ITPR, intertympanic pneumatic recess; ME, middle ear; MPC, median pharyngeal canal; OPR, otoccipital pneumatic recess; PHI, pharyngeal intersection; PHT, pharyngotympanic tubes; PP, posterior pre‐parietal process; RE, *recessus epitubaricus*; RPR, rhomboidal recess; SI, siphonium; TM, tympanic membrane. Scale bar = 10 mm.

The paratympanic sinuses are pneumatic cavities which link the tympanic membrane, the inner and middle ear, and the pharynx (Figures 8, 9, 10). They are well‐preserved in the skull of *E. lerichei*, and are similar to other gavialoids in terms of overall volume and shape, especially those of *Tomistoma schlegelii* (Kuzmin et al., 2021) and *Gryposuchus neogaeus* (Bona et al., 2015), as well as *Portugalosuchus azenhae* (Puértolas‐Pascual et al., 2023).

From the median pharyngeal foramen, the median pharyngeal canal (MPC) ascends along the basisphenoid‐basioccipital suture. The length of the MPC is one‐quarter of the total height of the paratympanic sinuses. Dorsally, the MPC enlarges anteroposteriorly and separates into two equidimensional branches in the basisphenoid and the basioccipital. This “pharyngeal intersection” forms a wide “U”‐shape in lateral view, a morphology that is also observed in both the extant genera *Tomistoma* and *Gavialis* (Figures 9 and 10), as well as in *Portugalosuchus azenhae* (Puértolas‐Pascual et al., 2023).

The anterior branch (or basisphenoid component) of the MPC widens to form the basisphenoid recess. The latter appears as two short lateral branches, which decrease in size dorsally into the *recessus epitubaricus*, a paired pneumatic lamina developed on each dorsal side of the basisphenoid. The *recessus epitubaricus* is not well‐developed ventrally and does not enter the pterygoid, as is also the case in extant gavialoids and most species of *Crocodylus* (Kuzmin et al., 2021; Perrichon et al., 2023b). In extant species, the *recessus epitubaricus* links the basisphenoid recess to each pharyngotympanic cavity (Figure 8). Unfortunately, due to the dorsoventral crushing of both prootics, none of the dorsal parts of the anterior pharyngeal canals are preserved, and we are not able to see their connection with the middle ear (Figure 8).

The posterior branch of the MPC is triangular in cross‐section and forms the basioccipital recess, which separates into two bulbous and elongate branches that are directed dorsolaterally. Each branch narrows to join the rhomboidal recess dorsolaterally. The rhomboidal recess is located at the interface between the basioccipital, the otoccipital, and the quadrate. The dorsal displacement of the basioccipital‐basisphenoid complex has a marked “step‐like” shape (at an ~90° angle) in the middle of the rhomboidal recess (Figure 8). The lateral pharyngotympanic (“Eustachian”) tubes are connected ventrally to the rhomboidal recess. Each tube shows a vertical course along the basisphenoid‐basioccipital suture, then shifts medially under the basisphenoid to join the pharynx. The lateral Eustachian foramina are positioned dorsally to the median pharyngeal foramen, nearly at the level of the posterior foramen for the cerebral carotid arteries. However, it is difficult to assess their exact size and position, due to the widening of the basisphenoid‐basioccipital suture.

The pharyngotympanic cavity, or middle ear sensu stricto, extends from the tympanic membrane to the fenestra ovalis of the inner ear. The left middle ear is deformed dorsoventrally, but the right one seems unaffected (Figure 8). Medially, the middle ear narrows and contacts the inner ear on the *fenestra ovalis*. Due to the breakage of the extracapsular buttress and the partial preservation of the *crista interfenestralis*, the limits of the sinus at this point are not well defined and it is not possible to distinguish its separation from the extracapsular portion of the inner ear. The columella is not preserved. The subordinated recesses of the middle ear are not well‐developed: anteriorly, the infundibular recess is present as a thin, dorsoventrally compressed canal that joins the subtympanic foramen. The prootic recess is absent or not preserved. Laterally, the quadrate recess is not expanded, meaning that the siphonium is directly connected to the pharyngotympanic cavity, as in the two extant gavialoid species (Figures 8, 9, 10).

**FIGURE 11:**
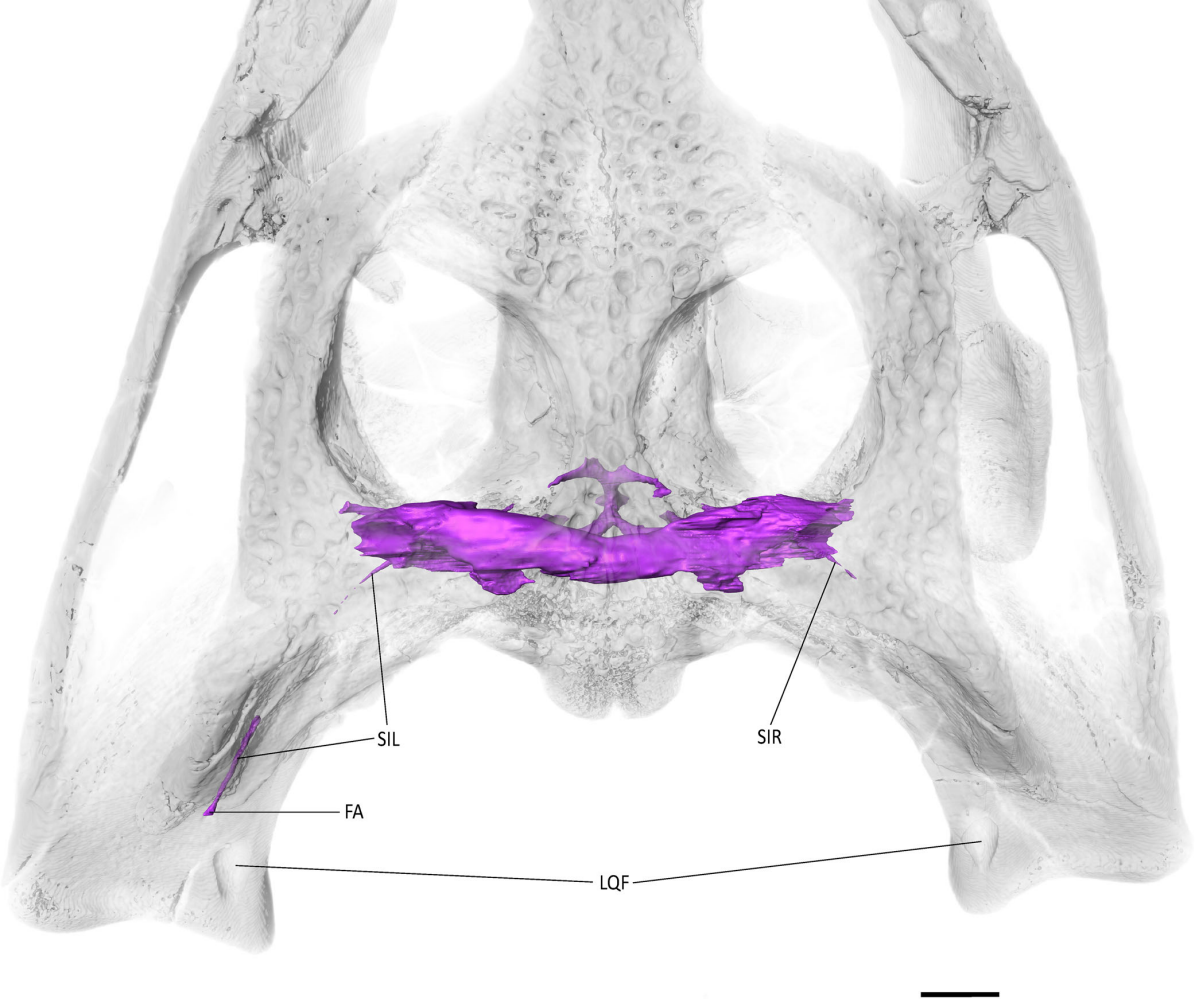
The paratympanic sinus system of *Eosuchus lerichei* (IRSNB R49) in dorsal view with the skullrendered transparent. FA, foramen aereum; LQF: Large quadrate foramen; SIL, left siphonium; SIR, right siphonium. Scale bar = 10 mm.

The siphonium exits the quadrate through the foramen aereum (Figures 8, 9, 10). The anterior parts of both siphonia are preserved. As a result of the CT‐scan data, we are able to confirm that the “foramen aereum” mentioned in previous descriptions of *E. lerichei*, which was described as “extremely enlarged” and considered an autapomorphy of the genus (Brochu, 2006; Delfino et al., 2005), does not correspond to the real foramen aereum. The latter corresponds to the posterior passage for the siphonium in extant crocodylians (Kuzmin et al., 2021). On the right side of the skull, the posterior end of the siphonium and the associated true foramen aereum are preserved and the siphonium of *E. lerichei* emerges through this “true” foramen aereum 10 mm anteriorly to the opening identified as the foramen aereum by Delfino et al. (2005) (Figure 11). The large quadrate foramen situated on the medial condyle of the quadrate is not actually linked to any canal: instead, it is only a depression on the quadrate surface (Figure 11). Consequently, this morphology still represents a diagnostic characteristic of at least *E. lerichei*, but the internal anatomy of the skull of *Eosuchus minor* needs to be studied to determine if it diagnoses the genus.

**FIGURE 12:**
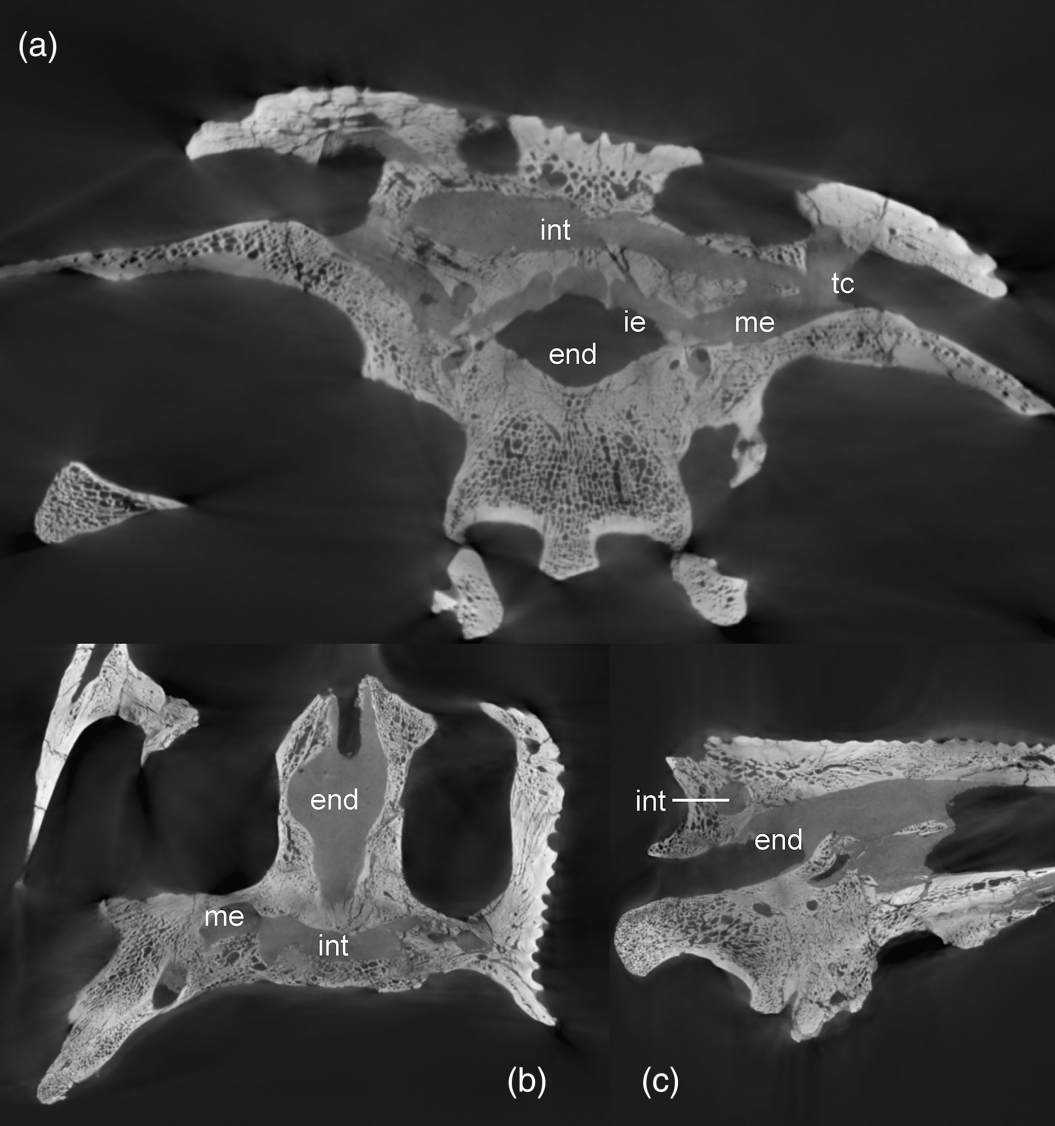
Internal views of the braincase of *Eosuchus lerichei* showing the morphology of the intertympanic pneumatic recess. (a) sagittal; (b) axial; and (c) coronal slices. END, brain endocast; IE, inner ear; INT, intertympanic sinus; ME, middle ear; TC, tympanic cavity.

The dorsomedial region of the middle ear is connected to the intertympanic recess through a large foramen that is delimited by the loop of the prootic buttress (Kuzmin et al., 2021; Perrichon et al., 2023a). This intertympanic recess is subcircular in cross‐section and occupies the supraoccipital from one tympanic cavity to another. The left part of the intertympanic recess is crushed dorsoventrally (Figure 12). This recess is moderately developed compared to most extant crocodylian species (Kuzmin et al., 2021). Its medial portion is constricted and slightly displaced posteriorly. Two small paired medial protrusions are directed dorsally and may correspond to the posterior pair of pre‐parietal processes (Figure 8; see also Perrichon et al., 2023a). There is no pneumatic development anteriorly: the sinus does not invade the parietal and does not form a parietal recess. Among extant crocodylians, this condition is only found in *Tomistoma schlegelii*, but it also characterizes a few extinct crocodyliforms, including *Gryposuchus* and *Portugalosuchus* (Bona et al., 2015; Perrichon et al., 2023a; Puértolas‐Pascual et al., 2023).

The lateral portion of the intertympanic recess is connected posteriorly to the otoccipital recess. This otoccipital recess is reduced, triangular‐shaped, and only present in the most dorsolateral part of the otoccipital (Figure 8). The moderate expansion of this recess is similar to the condition in *Tomistoma schlegelii* (Figure 10) and *Gryposuchus neogaeus* (Bona, et al., 2015).

### 4.5 Nasal cavity and other associated structures

**FIGURE 13:**
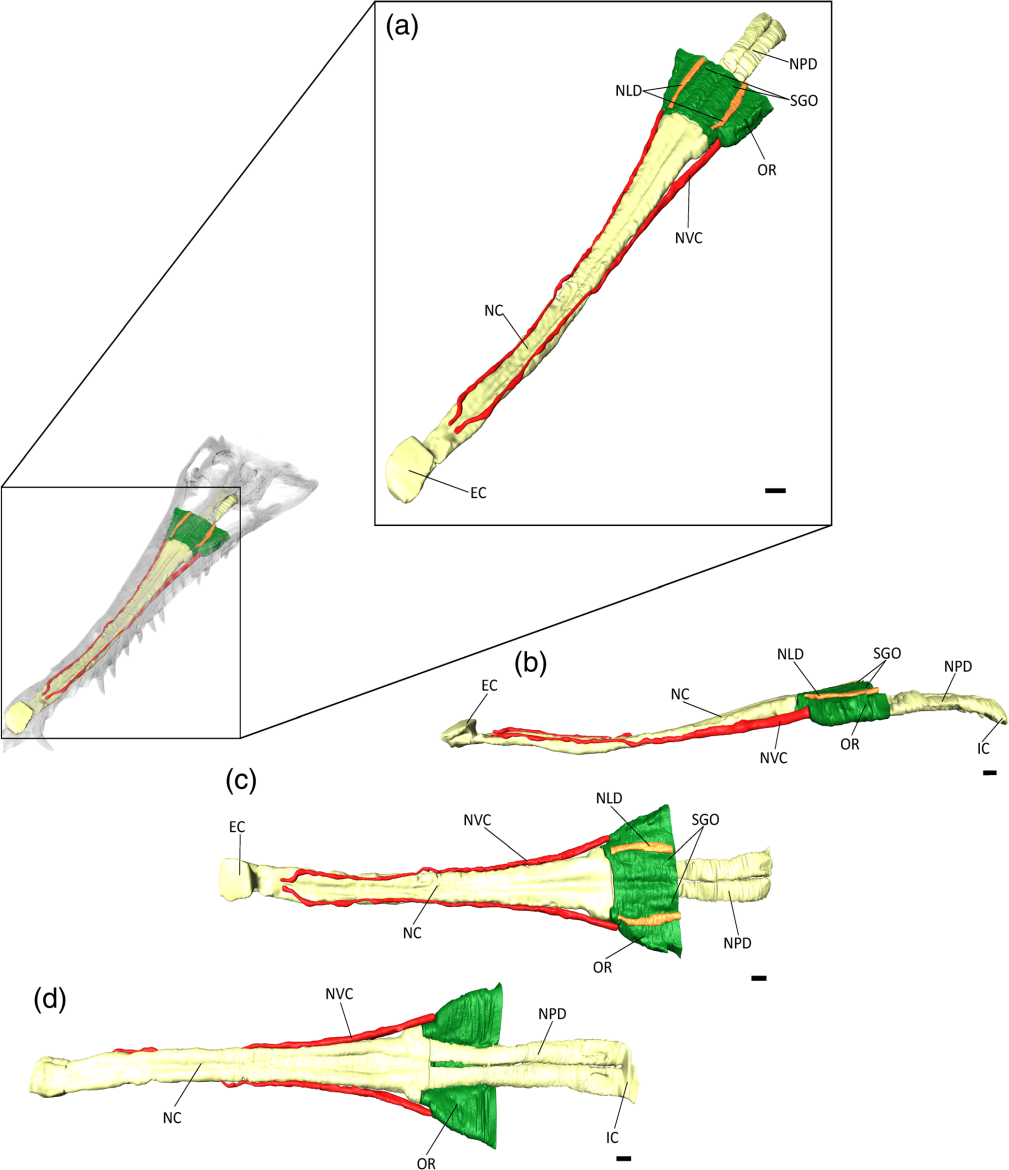
Olfactory region and nasal cavities of *Eosuchus lerichei* (IRSNB R49) in (a) anterior oblique, (b) lateral, (c) dorsal, and (d) ventral view. EC, external choana; IC, internal choana; NC, nasal cavity; NLD, nasolacrimal duct; NPD, nasopharyngeal duct; NVC, neurovascular canals; OR, olfactory region; SGO, potential salt gland osteological correlates. Scale bars = 10 mm.

The nasal cavity of *E. lerichei* generally reflects that of previously evaluated gavialoids, in which it extends from the premaxilla posteriorly, toward the basicranium (Pierce et al., 2017; Serrano‐Martínez et al., 2021; Burke & Mannion, 2023; Figure 13). At the premaxilla, the nasal cavity protrudes through the external naris, resulting in a similar morphology to both extant and fossil gavialoids. Throughout the premaxillary and maxillary bones, the nasal cavity reflects the shape of the rostrum, expanding to form the olfactory region anterior to the orbits. Neurovascular canals originate from the paranasal sinus and run anteroposteriorly, orientated laterally to the nasal cavity. These canals converge on the dorsal surface of the nasal cavity but do not meet (Figure 13c), just as in both extant gavialoids (Burke & Mannion, 2023).

As the nasal cavity gradually expands toward the orbits, the olfactory region forms, consisting of the paranasal sinus and the nasopharyngeal ducts. The paranasal sinus expands dorsolaterally, whereas the nasopharyngeal ducts are adjoined to the nasal cavity, bifurcating into two ducts posterior to the expansion of the paranasal sinus (Figure 13d). The nasopharyngeal ducts remain bifurcated, which differs from extant gavialoids, wherein the nasopharyngeal ducts merge into one duct toward the internal choana (Burke & Mannion, 2023). Each nasopharyngeal duct is also much more laterally expansive in *E. lerichei* than in extant gavialoids, such that its width is comparable to that of the maximum cerebrum width, as in *Portugalosuchus azenhae* (see Supplementary Material Table 1). By contrast, the nasopharyngeal ducts of extant gavialoids are approximately half the size of their maximum cerebrum width (see Supplementary Material Table 1). As in *Tomistoma schlegelii*, the entirety of the paranasal sinus protrudes over the nasopharyngeal duct in *E. lerichei* (Figure 13), which differs from *Gavialis gangeticus* and “*Tomistoma*” *dowsoni*, in which the paranasal sinus only partially protrudes over the nasopharyngeal duct, which is still visible in lateral view (Burke & Mannion, 2023). The dorsal surface of the olfactory region is characterized by two channel‐like nasolacrimal ducts that run parallel anteroposteriorly in *E. lerichei*, similar to the morphology in *Tomistoma schlegelii* (Burke & Mannion, 2023). The expansion of the olfactory region results in concave depressions on the internal surface of the prefrontal and lacrimal bones, which have been previously noted in several species of thalattosuchian crocodyliforms and interpreted therein as osteological correlates of salt glands (Cowgill et al., 2023).

### 4.6 Phylogenetic results

**FIGURE 14:**
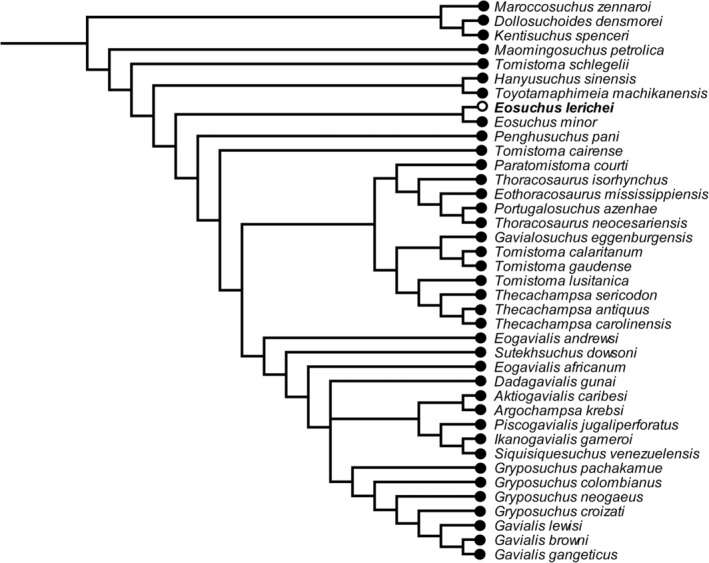
Strict consensus tree topology of Gavialoidea under equal weighting. *Eosuchus lerichei* is emboldened and *Melitosaurus champsoides* was pruned *a posterori*.

**FIGURE 15:**
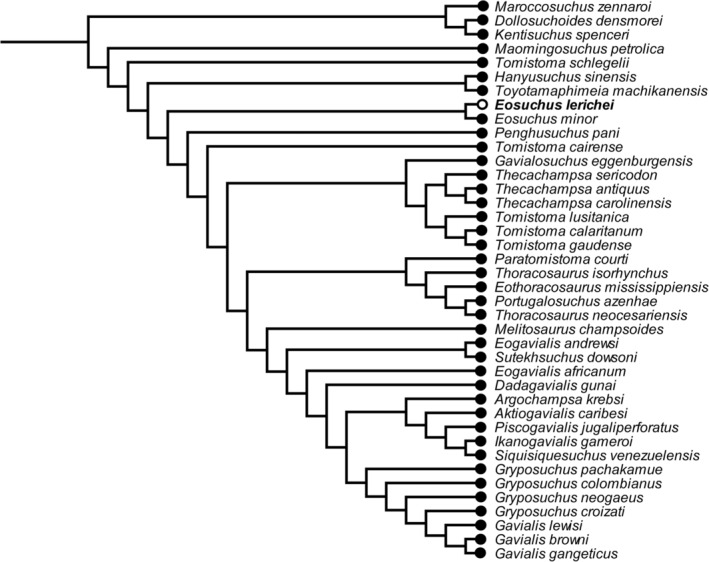
Strict consensus tree topology of Gavialoidea under extended implied weighting (*k* = 12). *Eosuchus lerichei* is emboldened.

Analysis with equal weights produced a large polytomy within Gavialoidea, with *Melitosaurus* identified as the most unstable taxon via the Pruned Trees option in TNT. Re‐analysis under equal weights, with *Melitosaurus* excluded a priori, results in 24 most parsimonious trees (MPTs), each with a length of 230,608.208 steps, a consistency index of 0.176 and a retention index of 0.656 (Figure 14). The extended implied weighting analysis resulted in nine MPTs, each with a length of 8483.886 steps, a consistency index of 0.173 and a retention index of 0.650 (Figure 15). *E. lerichei* is recovered as the sister taxon to *Eosuchus minor* in both sets of analyses, supported by two unambiguous synapomorphies: ratio of anteroposterior snout length to total skull length of 0.654–0.657 (character 1); and long axis of the posterior pterygoid process directed dorsoventrally (character 136: state 0). The global relationships of major clades relative to one another is unchanged relative to previous iterations of this matrix, with gavialoids recovered within Longirostres, which is recovered as the sister group to Crocodyloidea, with Alligatoroidea in a more “basal” position (Boerman et al., 2023; Burke et al., 2024; Puértolas‐Pascual et al., 2023; Rio & Mannion, 2021).

Within Gavialoidea, the tree topology in both analyses is relatively consistent with previous studies that have utilized this data matrix (see Burke et al., 2024). One notable difference concerns the position of *Eosuchus*, with the extended implied analysis recovering this taxon in a more “basal” position within Gavialoidea than in previous analyses, which is now congruent with the results of the equal weighting analysis (Figures 14 and 15). Under both weighting strategies, *Eosuchus* is not recovered as part of a “thoracosaur” grouping. However, “thoracosaurs” do form a monophyletic group, here comprising *Paratomistoma courti*, *Thoracosaurus isorhynchus*, *Eothoracosaurus mississippiensis*, *Portugalosuchus azenhae*, and *Thoracosaurus neocesariensis*, although the position of the clade varies slightly between the two analyses (Figures 14 and 15).

### 4.7 External anatomical comparisons with *Eosuchus minor*, “thoracosaurs” and other gavialoids

Although we focus on describing the internal cranial anatomy of *E. lerichei* in this contribution, newly observed and existing data on the external anatomy of this species also has a bearing on its phylogenetic placement. As such, here we make comparisons between the external anatomy of *E. lerichei* and other gavialoids, including species recovered as “thoracosaurs” (namely *Eothoracosaurus*, *Paratomistoma*, *Portugalosuchus*, and *Thoracosaurus*) in our phylogenetic analyses.

In both species of *Eosuchus*, the penultimate premaxillary alveolus is the largest. *Hanyusuchus sinensis*, *Maroccosuchus zennaroi*, and *Toyotamaphimeia machikanensis* are the only other gavialoids to share this morphology with *Eosuchus* (Brochu, 2006; Iijima et al., 2022; Iijima & Kobayashi, 2019; Jouve et al., 2015). In *Eothoracosaurus* and *Thoracosaurus isorhynchus*, the antepenultimate premaxillary alveolus is equidimensional to that of the penultimate alveolus, which is similar to the condition in extant *Gavialis* and *Tomistoma* (Brochu, 2004; Jouve et al., 2015; Piveteau, 1927; Rio & Mannion, 2021; Salas‐Gismondi et al., 2015, 2016). Additionally, the penultimate premaxillary alveolus of *E. osuchus lerichei* is positioned posteromedial to the antepenultimate alveolus, similar to the morphology of most gavialoids, with the exception of *Aktiogavialis caribesi*, *Eosuchus minor*, *Eothoracosaurus*, *and Thecachampsa*, in which the penultimate premaxillary alveolus is positioned posterolateral to the antepenultimate alveolus (Brochu, 2004, 2006; Jouve et al., 2015; Rio & Mannion, 2021). In *E. lerichei*, the posterior process of the premaxilla extends on the palate up to the level of two maxillary alveoli, as in *Eothoracosaurus* (Brochu, 2004; Jouve, 2004; Jouve et al., 2008; Rio & Mannion, 2021). This contrasts with *Eosuchus minor*, wherein the premaxilla extends up to the level of one maxillary alveolus, and *Thoracosaurus isorhynchus*, in which the premaxilla extends up to three maxillary alveoli. The premaxilla‐maxilla suture in *Eosuchus*, *Hanyusuchus*, *Maomingosuchus*, “*Tomistoma*” *dowsoni*, *Tomistoma schlegelii* and *Toyotamaphimeia machikanensis* is posteriorly bowed, with two or more apices, whereas there is only one acute apex in all other gavialoids (Brochu, 2006; Groh et al., 2020; Rio & Mannion, 2021).

In nearly all gavialoids, the shape of the maxillary toothrow posterior to the first six alveoli is laterally concave (Brochu, 1997; Clark, 1994; Rio & Mannion, 2021). However, in *Eothoracosaurus*, *Portugalosuchus*, *Maroccosuchus zennaroi* and “*Tomistoma*” *gaudense*, this region is laterally convex or straight instead. In *E. lerichei*, the maxillary alveoli are positioned ventrally or at the same level as the maxillary palate separating the toothrows. This feature is common in early diverging gavialoids, including *Thoracosaurus isorhynchus*, as well as in *Tomistoma schlegelii*, whereas the alveoli are positioned dorsal to the maxillary palate in *Eosuchus minor* and *Eothoracosaurus* (Brochu, 2004, 2006; Hua & Jouve, 2004; Jouve, 2016; Rio & Mannion, 2021).

**FIGURE 16:**
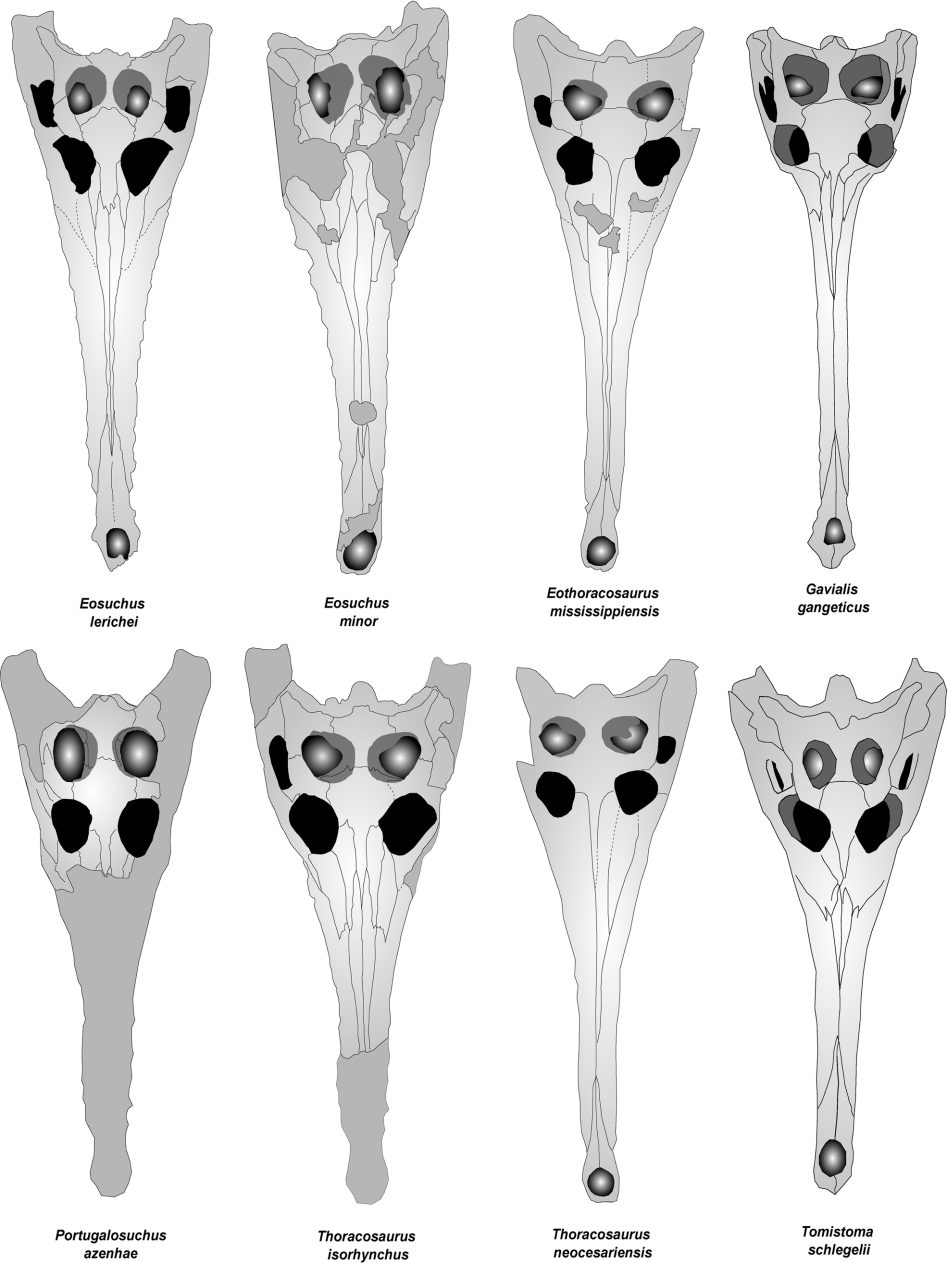
Line drawings of the skulls of *Eosuchus lerichei* and *Eosuchus minor*, as well as the “thoracosaurs” *Eothoracosaurus mississippiensis*, *Portugalosuchus azenhae*, *Thoracosaurus isorhynchus*, and *Thoracosaurus neocesariensis*, and extant gavialoids *Gavialis gangeticus* and *Tomistoma schlegelii* in dorsal view. Not drawn to scale.

*E. lerichei* is the only gavialoid in which the frontal forms a broad sutural contact with the nasals (Figure 16). In all other gavialoids, the frontal forms an acute V‐shape that extends anteriorly into the posterior margins of the nasals (Brochu, 2011; Rio & Mannion, 2021; Salas‐Gismondi et al., 2015). The anterior tip of the frontal is positioned posteriorly relative to the anterior tip of the prefrontal in *Eosuchus* (Jouve, 2004; Jouve et al., 2008), similar to the morphology in *Tomistoma schlegelii, Kentisuchus spenceri*, *Dollosuchoides densmorei*, *Maroccosuchus zennaroi, Maomingosuchus petrolica*, and *Thoracosaurus isorhynchus* (Brochu, 2007; Jouve et al., 2015; Shan et al., 2017). In most other gavialoids, including *Gavialis*, the tip of the frontal is positioned anterior to the anterior prefrontal margin. In *Eosuchus*, the anterior extent of the jugal is positioned anterior to that of the frontal, which is also exhibited in *Thoracosaurus isorhynchus* and *Tomistoma schlegelii*. By contrast, in some gavialoids, including *Eothoracosaurus* and *Portugalosuchus*, the anterior extent of the jugal is positioned posteriorly to the tip of the frontal (Brochu, 2004; Jouve, 2004, 2016; Jouve et al., 2008; Piveteau, 1927; Puértolas‐Pascual et al., 2023; Rio & Mannion, 2021).

The postorbital bar in *Eosuchus* is positioned posteriorly to the posterior extent of the maxilla (Brochu, 2011; Rio & Mannion, 2021). This morphology is seen in almost all gavialoids, including *Eothoracosaurus* and *Thoracosaurus*, with the exception of *Gavialis*, *Dadagavialis*, and *Portugalosuchus*, in which it is positioned anteriorly to the posterior extent of the maxilla (Brochu, 2004; Puértolas‐Pascual et al., 2023; Salas‐Gismondi et al., 2016). Both species of *Eosuchus* lack a posterodorsal jugal foramen at the base of the postorbital bar. The lack of a posterodorsal jugal foramen, or the possession of only a small foramen, is commonly seen in early diverging gavialoids as well as *Tomistoma schlegelii* (Rio & Mannion, 2021). However, a large foramen characterizes *Portugalosuchus* and *Thoracosaurus isorhynchus*, wherein its diameter is equal to or greater than that of half the minimum jugal arch width (Jouve, 2016; Piveteau, 1927; Puértolas‐Pascual et al., 2023; Rio & Mannion, 2021).

The dorsal profile of the jugal is straight and continuous with the dorsal margin of the lower temporal bar in both species of *Eosuchus*. By contrast, in all “thoracosaurs” for which the jugal is preserved, the dorsal profile slopes posteroventrally, gradually descending into the lower temporal bar (Brochu, 1997; Jouve, 2016; Jouve et al., 2006; Lee & Yates, 2018; Piveteau, 1927; Puértolas‐Pascual et al., 2023; Rio & Mannion, 2021). Most gavialoids do not possess a ventrolateral sulcus on the jugal and maxilla, including *Eosuchus* and “thoracosaurs,” with the exception of *Thoracosaurus isorhynchus* (Rio & Mannion, 2021). The sulcus is present, however, in *Eogavialis africanum*, *Gavialis gangeticus*, *Gavialis lewisi*, and *Gryposuchus colombianus* (Andrews, 1906; Kraus, 1998; Langston & Gasparini, 1997; Lull, 1944; Rio & Mannion, 2021; Wu et al., 1996, 2001).

The dorsomedial margin of the orbit is flush with the skull surface in *Eosuchus, Penghusuchus, Piscogavialis*, and most “thoracosaurs” (Figure 16). In all other gavialoids, including *Thoracosaurus isorhynchus*, the dorsomedial margin of the orbit is either upturned or projects into the orbit (Brochu, 1997, 2004; Iijima & Kobayashi, 2019; Kraus, 1998; Piveteau, 1927; Puértolas‐Pascual et al., 2023; Rio & Mannion, 2021).

*E. lerichei* has a frontoparietal suture that does not intersect the supratemporal fenestrae, and the postorbital‐parietal contact is fully exposed on the skull table (Figure 16). This morphology is exhibited in many gavialoids; however, the frontoparietal suture incipiently contacts the supratemporal fenestrae in *Eosuchus minor, Aktiogavialis caribesi, Gavialis gangeticus, Gryposuchus colombianus, Ikanogavialis gameroi*, and *Thecachampsa sericodon*, in which the postorbital‐parietal suture is slightly exposed on the skull table (Brochu, 1997, 2006; Langston & Gasparini, 1997; Rio & Mannion, 2021; Salas‐Gismondi et al., 2019; Sill, 1970; Weems, 2018), and the frontoparietal suture deeply intersects the supratemporal fenestrae in *Eothoracosaurus*, *Portugalosuchus* and *Thoracosaurus*, such that the postorbital‐parietal suture is not exposed on the skull table (Brochu, 2004; Piveteau, 1927; Puértolas‐Pascual et al., 2023). In both species of *Eosuchus*, the frontoparietal suture is concavo‐convex, whereas the suture is straight in “thoracosaurs” (Brochu, 1997, 2004; Piveteau, 1927; Puértolas‐Pascual et al., 2023; Rio & Mannion, 2021).

The squamosal‐parietal suture intersects the dorsal margin of the orbitotemporal foramen in *E. lerichei*, and there is a large medial fossa on the posterior wall. This morphology also characterizes *Tomistoma schlegelii*, as well as early diverging gavialoids such as *Maomingosuchus petrolica* and *Maroccosuchus zennaroi* (Jouve et al., 2015; Rio & Mannion, 2021; Shan et al., 2017). Comparatively, there is little to no development of a fossa medial to the orbitotemporal foramen in “thoracosaurs,” and the squamosal‐parietal suture passes medially to the foramen (Mateus et al., 2019; Rio & Mannion, 2021).

The posterolateral edges of the skull table of *E. lerichei* are directed ventrolaterally from the sagittal axis. The same morphology is exhibited in *Eothoracosaurus* and *Thoracosaurus*, as well as *Gavialis* and gryposuchines (Barrios, 2011; Brochu, 1997; Cidade et al., 2017; Jouve, 2016; Rio & Mannion, 2021). In *Eosuchus minor, Portugalosuchus azenhae*, and *Tomistoma schlegelii*, the posterolateral edges are planar across the entire length of the skull table (Brochu, 2006; Puértolas‐Pascual et al., 2023; Rio & Mannion, 2021).

In *E. lerichei*, the quadratojugal does not reach the dorsal angle of the infratemporal fenestra (Figure 3) (Brochu, 1997; Buscalioni et al., 1992; Rio & Mannion, 2021). In all “thoracosaurs,” as well as *Eogavialis africanum*, *Eosuchus minor*, *Gavialis gangeticus, Gavialis lewisi, Gryposuchus colombianus, Gryposuchus pachakamue, Kentisuchus spenceri, Maomingosuchus petrolica*, and *Tomistoma schlegelli*, the quadratojugal reaches the dorsal angle of the infratemporal fenestra (Andrews, 1906; Buscalioni et al., 1992; Langston & Gasparini, 1997; Brochu, 1997, 2006, 2007; Salas‐Gismondi et al., 2016; Shan et al., 2017; Rio & Mannion, 2021).

The exoccipitals contact the basioccipital tubera in both species of *Eosuchus*; however, this is not the case in several gavialoids, such as *Gavialosuchus*, *Hanyusuchus*, *Kentisuchus*, *Maomingosuchus*, *Maroccosuchus*, *Paratomistoma*, *Penghusuchus*, *Portugalosuchus, Thecachampsa, Thoracosaurus, Tomistoma schlegelii*, and *Toyotamaphimeia* (Brochu, 1997, 2004; Brochu and Gingerich, 2000; Clark, 1994; Iijima & Kobayashi, 2019; Iijima et al., 2022; Jouve et al., 2015; Norell, 1988; Piveteau, 1927; Puértolas‐Pascual et al., 2023; Rio & Mannion, 2021; Shan et al., 2017; Toula & Kail, 1885; Weems, 2018). Additionally, in *Eosuchus*, as well as *Eothoracosaurus*, *Thoracosaurus isorhynchus*, and *Tomistoma schlegelii*, the exoccipitals do not have a posteroventral inclination and they are therefore not visible in dorsal view. Conversely, *Portugalosuchus* and *Thoracosaurus neocesariensis* reflect the same morphology as *Gavialis*, in which the exoccipitals are posteroventrally inclined (Brochu, 2004; Hua & Jouve, 2004; Jouve et al., 2008; Piveteau, 1927; Puértolas‐Pascual et al., 2023).

The anterior process of the palatine of both species of *Eosuchus* is rounded, similar to *Hanyusuchus*, *Maomingosuchus*, *Maroccosuchus*, and *Toyotamaphimeia*; by contrast, most gavialoids possess a wedge‐shaped anterior process, including *Eothoracosaurus* and *Thoracosaurus isorhynchus* (Brochu, 1997, 2004, 2006; Iijima et al., 2022; Iijima & Kobayashi, 2019; Jouve et al., 2015; Rio & Mannion, 2021; Shan et al., 2017). Similarly, *Eosuchus* possesses an invagination of the anterior process, as in *Gavialis*, whereas this is absent in *Eothoracosaurus, Thoracosaurus*, and *Tomistoma schlegelii* (Brochu, 1997, 2004; Delfino & De Vos, 2010; Rio & Mannion, 2021). In *E. lerichei* and all “thoracosaurs,” the anterior process of the palatine is positioned anterior to the suborbital fenestra margin, at the level of more than two full alveoli. In *Eosuchus minor*, the anterior process of the palatine is also positioned anteriorly to the suborbital fenestra; however, it extends only to the level of less than two full alveoli (Brochu, 1997, 2004; Puértolas‐Pascual et al., 2023; Rio & Mannion, 2021; Willis, 1993).

In *E. lerichei*, the maxilla‐palatine suture intersects the suborbital fenestra at the anterior corner, as in *Thoracosaurus isorhynchus*, *Gavialis*, and many other late–diverging gavialoids. In *Eosuchus minor*, the maxilla‐palatine suture intersects the suborbital fenestra at the anteromedial margin, as in *Eothoracosaurus* and *Tomistoma schlegelii* (Brochu, 2004, 2006; Brochu & Storrs, 2012; Piveteau, 1927; Rio & Mannion, 2021).

*E. lerichei* and all “thoracosaurs” possess an ectopterygoid with an anterior extent that extends to the level of two or fewer maxillary alveoli (Jouve, 2016; Lee & Yates, 2018; Rio & Mannion, 2021). Comparatively, the anterior extent of the ectopterygoid in *Eosuchus minor* extends further than two full alveoli, as in *Dollosuchoides densmorei*, *Eogavialis africanum*, *Gavialis lewisi*, *Gryposuchus colombianus*, *Piscogavialis*, and “*Tomistoma*” *dowsoni* (Brochu, 2006; Salas‐Gismondi et al., 2016, Salas‐Gismondi et al., 2022; Rio & Mannion, 2021; Burke et al., 2024). *E. lerichei* has an acute posterior process of the ectopterygoid on the medial jugal surface, that terminates before the posterior margin of the postorbital bar (Jouve, 2004, 2016; Norell, 1989; Rio & Mannion, 2021). In *Eosuchus minor, Gavialis*, and all “thoracosaurs” in which the ectopterygoid and jugal are preserved, the acute process of the ectopterygoid extends beyond the posterior margin of the postorbital bar (Brochu, 2004, 2006; Piveteau, 1927; Puértolas‐Pascual et al., 2023).

*E. lerichei* has a septum present within the choanae, as in *Tomistoma schlegelii*, whereas *Eosuchus minor*, all “thoracosaurs,” and *Gavialis gangeticus* lack this feature (Brochu, 1997, 2004, 2006; Groh et al., 2020; Piveteau, 1927; Puértolas‐Pascual et al., 2023).

The anteriormost dentary tooth in *E. lerichei* projects sub‐horizontally at an angle of less than 30° from the ventral margin of the dentary. All other gavialoids, including *Eosuchus minor*, “thoracosaurs” and the extant species, have an anteriormost dentary tooth that projects at an angle greater than 30° (Figure 4) (Brochu, 1997; Burke et al., 2024; Piveteau, 1927; Rio & Mannion, 2021).

### 4.8 Taxonomic status of specimens previously assigned to *E. lerichei*

Currently, *E. lerichei* is not known from other localities in Europe, but it has been identified in the Paleogene of the USA (Weems, 1999) and tentatively in Thailand (Ducrocq et al., 1995), which would greatly extend the spatial distribution of this species. Here we discuss these referrals.

Weems (1999) referred several specimens (premaxillae, teeth, osteoderms) from the lower Eocene Nanjemoy Formation at the Fisher/Sullivan site in Virginia, USA, to *E. lerichei*. The premaxillae surround the external naris, and each element possesses five alveoli. Of the three most posterior alveoli, the penultimate premaxillary alveolus is the largest, as in *E. lerichei*. However, this morphology is also seen in *Eosuchus minor*, as well as several other gavialoids, for example, *Hanyusuchus sinensis*, *Maroccosuchus zennaroi*, and *Toyotamaphimeia machikanensis* (Brochu, 2006; Iijima et al., 2018; Iijima et al., 2022; Jouve et al., 2015). Weems (1999) noted that *Eosuchus minor* (at the time referred to as *Thecachampsa minor*) did not notably differ anatomically from *E. lerichei*, and, as such, we consider it most likely that these remains are assignable to *Eosuchus minor* instead.

Ducrocq et al. (1995) listed the presence of cf. *E. lerichei* in their faunal list of the upper Eocene Krabi Basin of southern Thailand, but provided no description or illustration of this material. Given that subsequent authors have identified the crocodylians *Maomingosuchus* and *Krabisuchus* from these deposits (Martin & Lauprasert, 2010; Shan et al., 2017), it is possible that this material might be referrable to one of those taxa instead. Pending its location and study, we consider it as Crocodylia indet.

Following our reassessment of these remains from the USA and Thailand, we restrict the known distribution of *E. lerichei* to its Belgian type locality.

## 5 DISCUSSION

### 5.1 Phylogenetic positions of *Eosuchus* and “thoracosaurs”

In its first inclusion in a phylogenetic analysis (Delfino et al., 2005), *Eosuchus* was nested in Gavialoidea, but separate from taxa recovered as “thoracosaurs”. In recent analyses, the phylogenetic position of *Eosuchus* relative to “thoracosaurs” has been labile, with support for both its exclusion and inclusion (see Boerman et al., 2023; Burke et al., 2024; Puértolas‐Pascual et al., 2023; Rio & Mannion, 2021). Following our updated scoring of *E. lerichei*, the genus *Eosuchus* is not recovered as closely related to any other species commonly referred to as a “thoracosaur” in either our equal or extended implied weights analysis. This has been driven by the scoring of additional characters based on CT scans, in combination with the rescoring of other characters. In this study, 98 out of 330 total characters were rescored for *E. lerichei*, five of which were inferred from the newly acquired CT data, with the remaining 92 changed based on observations of the external anatomy.

**FIGURE 17:**
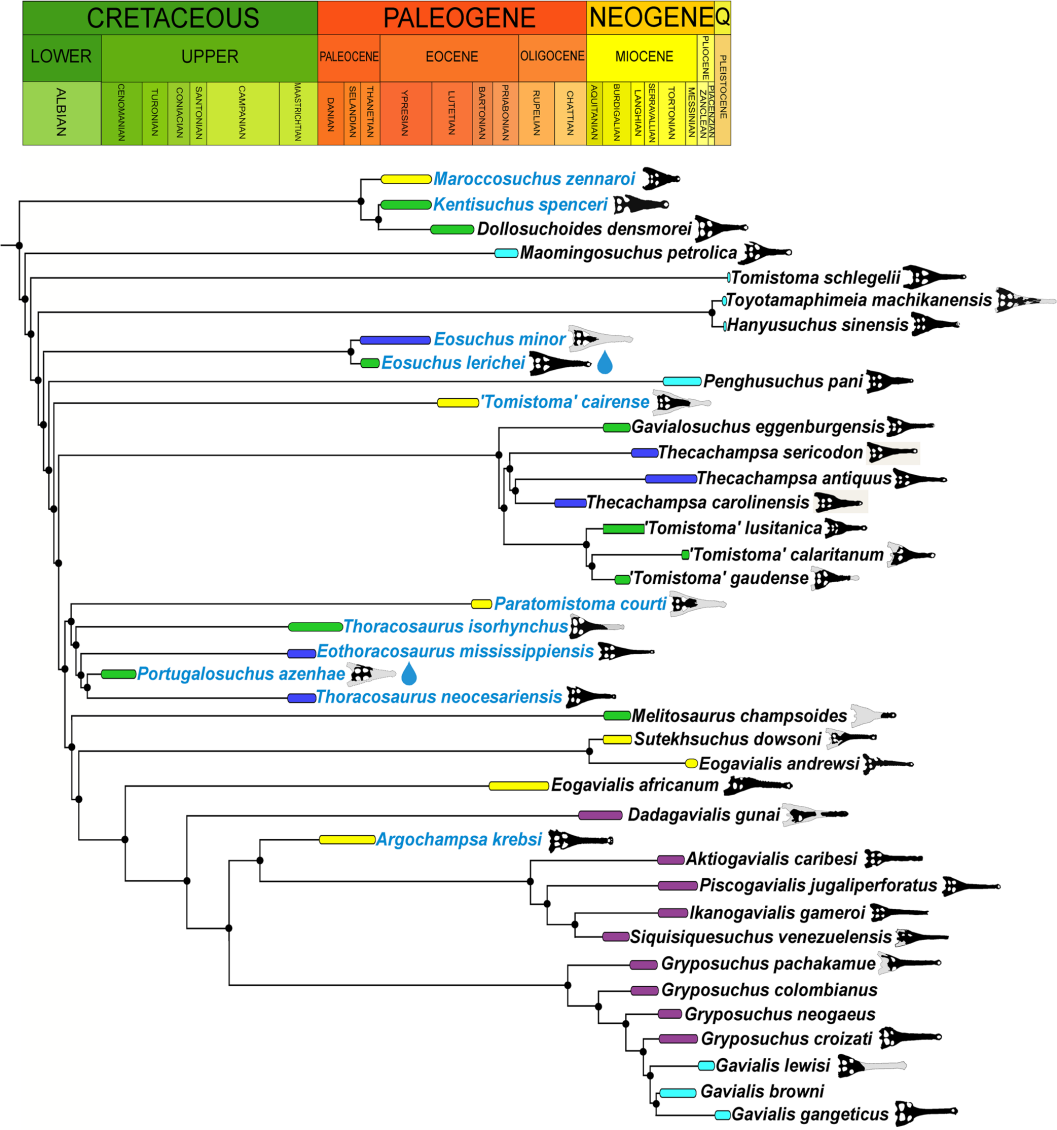
Time calibrated phylogeny of Gavialoidea based on the extended implied weighting analysis. Species names in blue indicate that they are found in marine deposits. Stratigraphic ranges (including temporal uncertainty) are color‐coded biogeographically: Africa = yellow, Asia (including India) = light blue, Europe = green, North America = dark blue, South America = purple. Teardrop symbol = evidence of osteological correlates for salt glands.

In broad agreement with the results of the phylogenetic analyses of Burke et al. (2024), both sets of our analyses recover a monophyletic group of “thoracosaurs,” consisting of the Late Cretaceous–early Paleocene European and North American taxa *Eothoracosaurus*, *Portugalosuchus*, and *Thoracosaurus*, as well as the middle Eocene north African genus *Paratomistoma* (Figure 17). Although these changes have not resolved the temporal incongruence of either *Eosuchus* or “thoracosaurs” being recovered as gavialids more closely related to *Gavialis* than to *Tomistoma* (see also discussion in Burke et al., 2024; Darlim et al., 2022; Lee & Yates, 2018), the placement of *Eosuchus* in a more “basal” position than previous iterations of this data matrix is at least in greater accordance with its early stratigraphic age (late Paleocene–early Eocene). Therefore, we suggest that both the incorporation and scoring of other morphological characters based on CT data, and the revision of character scorings of other early gavialoid taxa more generally, may increasingly lead to a more temporally congruent phylogenetic tree. Thus far, the former has only been possible for *Portugalosuchus* (Puértolas‐Pascual et al., 2023) and now *E. lerichei*.

### 5.2 Phylogenetic significance of endocranial structures

**FIGURE 18:**
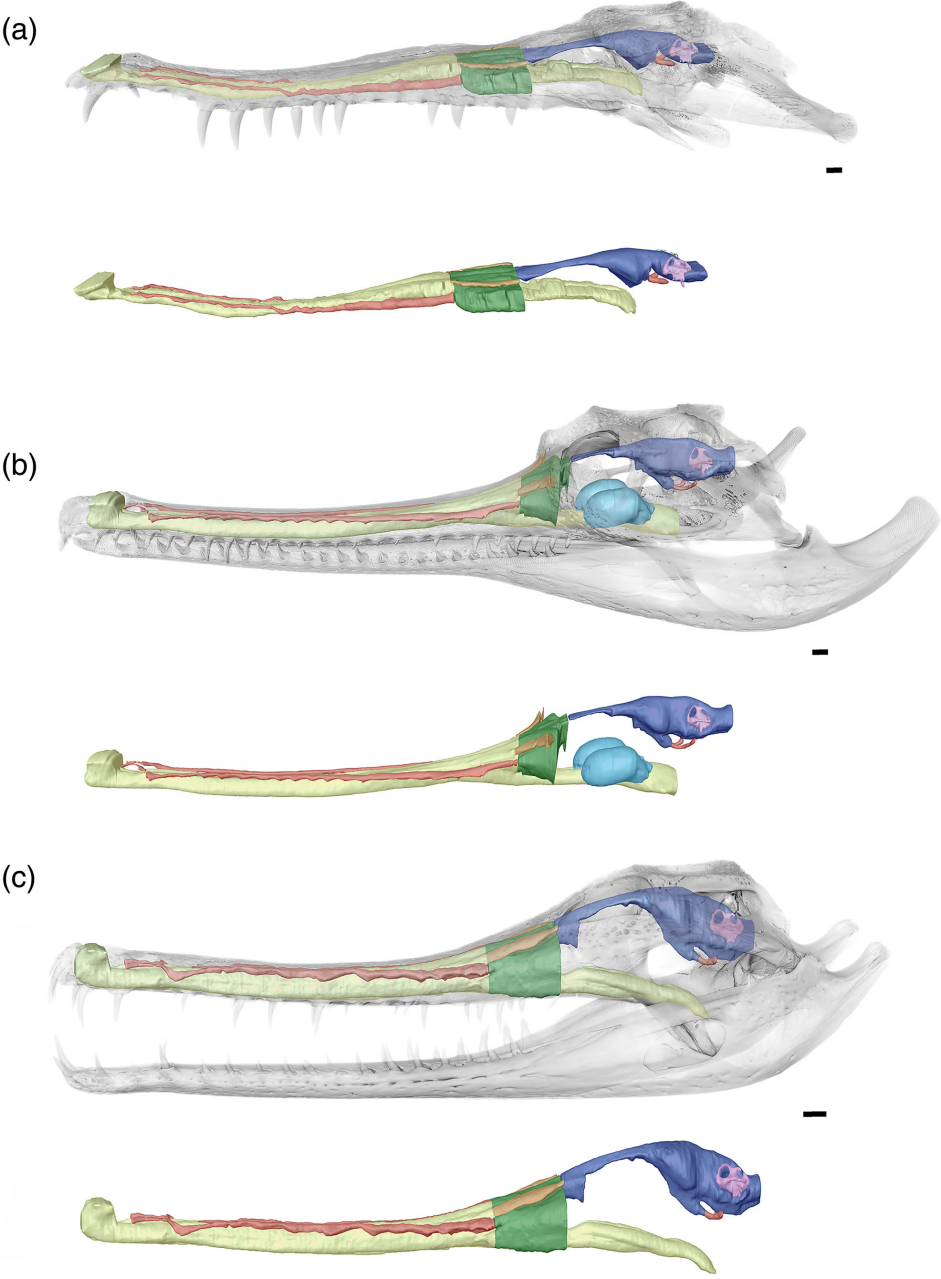
(a) The neuroanatomy of gavialoids in left lateral view, comparing (a) *Eosuchus lerichei*; b) *Gavialis gangeticus*; and (c) *Tomistoma schlegelii*. Scale bars = 10 mm.

**FIGURE 19:**
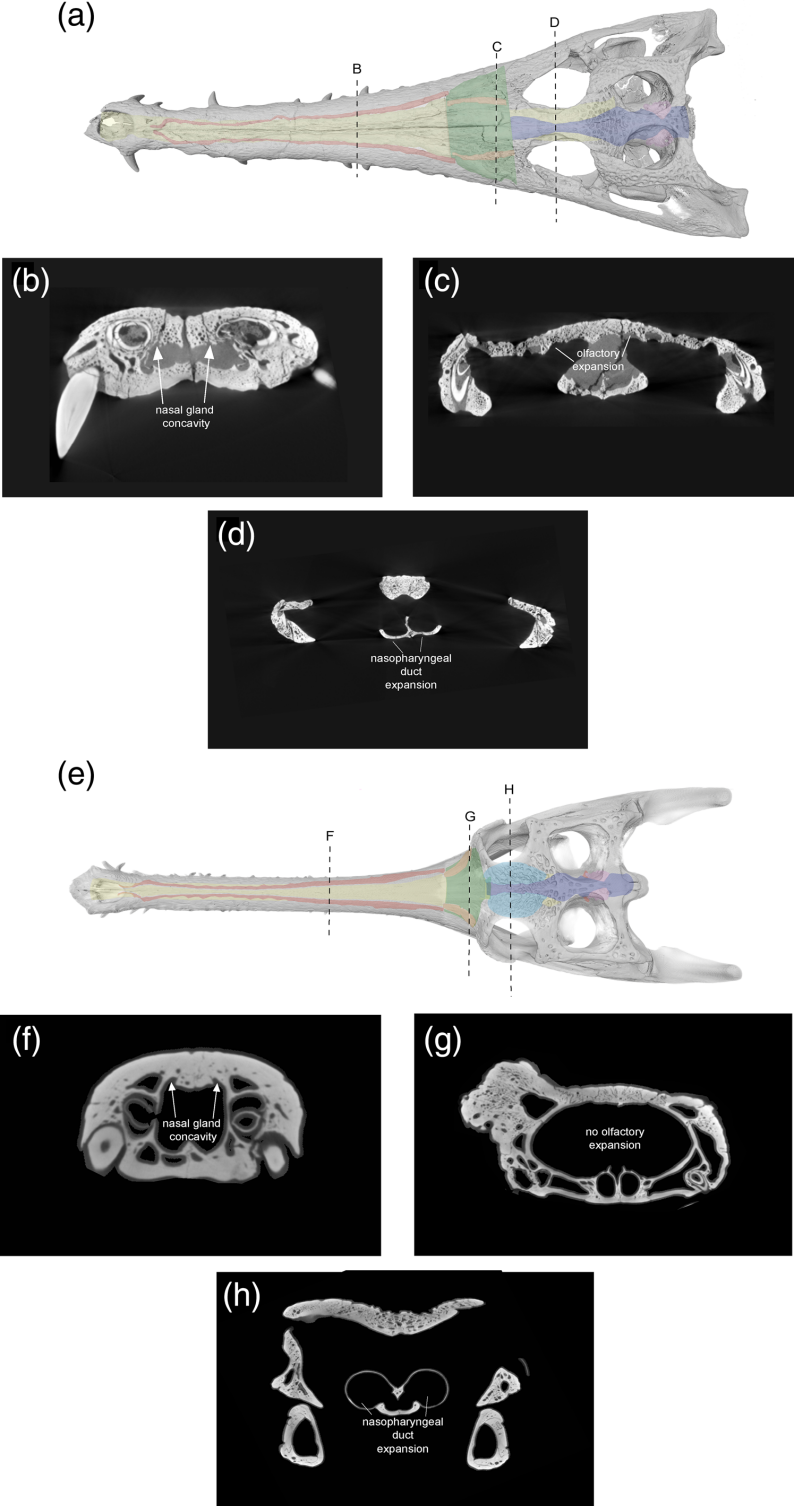
(a) The endocranial anatomy and skull of *Eosuchus lerichei* in dorsal view, with (b) showing the position of the nasal gland concavities, and (c) the concave depressions on the internal surface of the prefrontals and lacrimals indicating the olfactory expansions and salt glands and (d) the expansion of the nasopharyngeal duct. (e) The endocranial anatomy and skull of *Gavialis gangeticus* with (f) showing the position of the nasal gland concavities and (g) the internal surface of the prefrontals and lacrimals of *Gavialis* showing no olfactory expansion and (h) the expansion of the nasopharyngeal duct.

*Gavialis gangeticus* possesses an egg‐shaped pterygoid bulla parallel to the nasopharyngeal duct (Figure 18; Martin and Bellairs, 1977; Pierce et al., 2017). This structure has also been documented in several extinct gavialoid species, namely *Gavialis lewisi* and *Gavialis bengawanicus* (Lull, 1944; Martin et al., 2012), *Eogavialis africanum* (Hecht & Malone, 1972), and *Hanyusuchus sinensis* (Iijima et al., 2022), as well as possibly in *Dadagavialis* and *Gryposuchus* too (Riff & Aguilera, 2008; Salas‐Gismondi et al., 2016, 2019). Although *Eosuchus* does not possess a pterygoid bulla, the nasopharyngeal duct is much more laterally expansive than in *Gavialis*, equating to the width of the cerebrum, whereas the nasopharyngeal duct is approximately half the width of the cerebrum width in *Gavialis* (see Supplementary Material Table 1). Additionally, the nasopharyngeal ducts of *Eosuchus* are bifurcated throughout, whereas in *Gavialis* they merge into one duct anterior to the pterygoid bulla (see Burke & Mannion, 2023: fig. 3c). A similar morphology characterizes the “thoracosaur” *Portugalosuchus*, which has a comparably laterally expansive nasopharyngeal duct that is bifurcated throughout (Puértolas‐Pascual et al., 2023). In *Gavialis*, the nasopharyngeal duct is enclosed by the palatines, separated from the pterygoid bulla at the anterior and posterior ends; however, in the medial and largest section of the bulla, there is no clear separation between the nasopharyngeal duct and the bulla (see Figure 19d,h). Therefore, the expansion of the nasopharyngeal duct in *Eosuchus*, could be an early form of the pterygoid bulla expansion (see Figure 12b). *Eogavialis gavialoides* (AMNH 5067) also shows evidence of an expansion from the palatines, similar to that seen in *E. lerichei*, and which could be interpreted as a pterygoid bulla in its earliest form, and *Dollosuchoides densmorei* has a slight lateral expansion, which has been previously referred to as a pterygoid bulla (Brochu, 2006: fig. 3d).

The basioccipital, basisphenoid, infundibular, and quadrate recesses are thin and tubular in *E. lerichei*, whereas the intertympanic and otoccipital recesses are mostly confined to the supraoccipital. This morphology is comparable to extant gavialoids, but is more similar to *Tomistoma schlegelii* than to *Gavialis gangeticus* with regards to the relative thickness of the anterior and posterior pneumatic canals, and the arrangement of the recesses (Figures 8, 9, 10, 11). The dorsal part of the pneumatic system is particularly reminiscent of the morphology of *Tomistoma schlegelii*: the subtympanic foramen is still present; the intertympanic recess is constricted dorsoventrally and anteroposteriorly; the otoccipital recess shows the same shape and expansion; and the parietal is not pneumatized. This last feature contrasts with the condition in *Gavialis gangeticus*, wherein the parietal is heavily pneumatized by a large sinus expansion. This common pneumatic morphology shared by *E. lerichei* and *Tomistoma schlegelii* is also found in *Portugalosuchus azenhae*, in which the pneumatic system also shows thin canals, an elongated middle ear, and reduced dorsal pneumatization (Puértolas‐Pascual et al., 2023). It can be found to a lesser extent in *Gryposuchus neogaeus*, which also lacks a parietal pneumatization (Bona et al., 2015). The repeated observation of an absence of parietal pneumatic recess in *Tomistoma schlegelii* and currently sampled fossil gavialoids (including *E. lerichei*) could mean that a reduction of dorsal sinus pneumaticity occurred early in the evolutionary history of gavialoids, with a reversal to increased pneumatization in *Gavialis gangeticus*.

### 5.3 Ecological inferences

**FIGURE 20:**
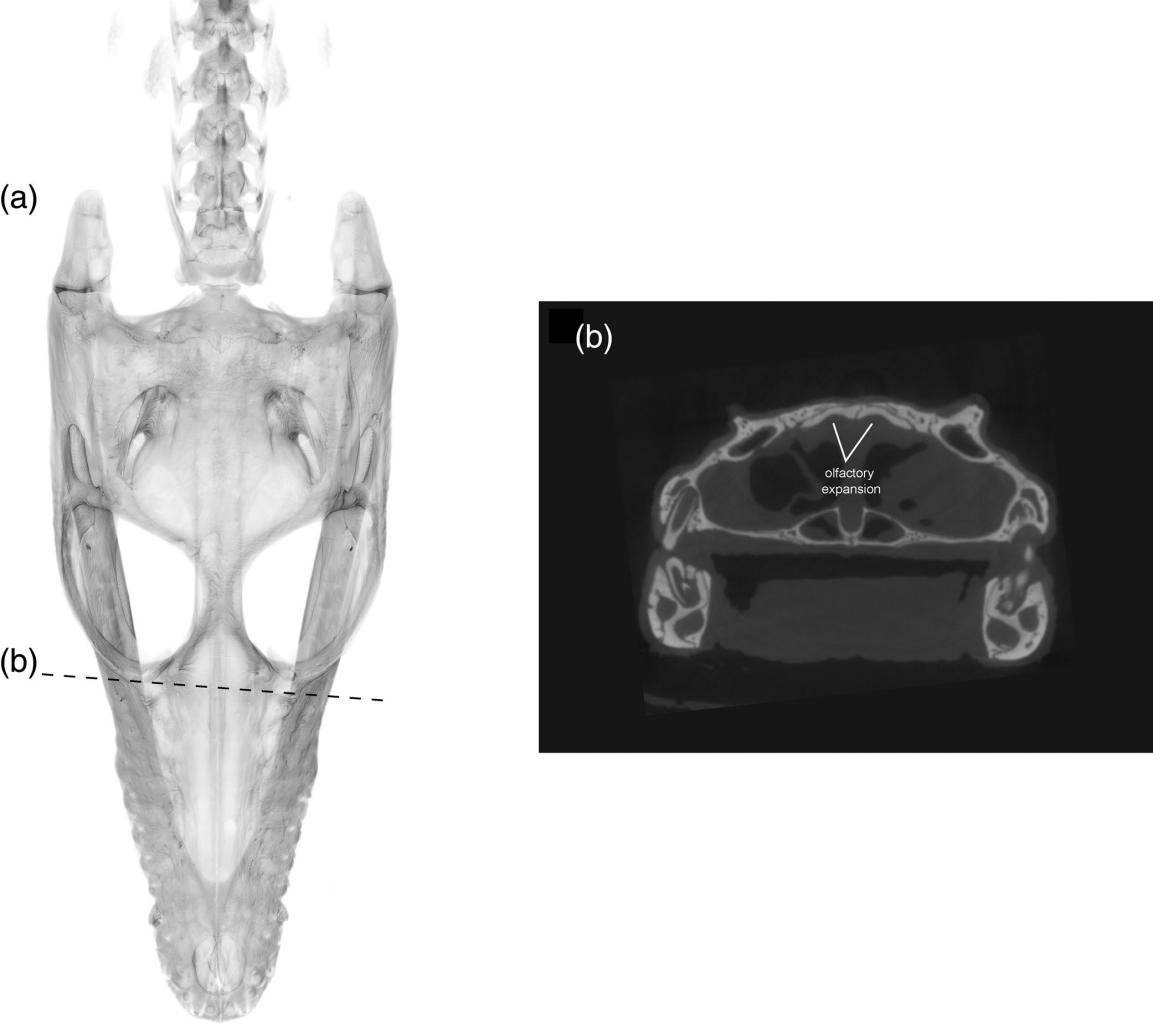
(a) Skull rendering of juvenile *Crocodylus porosus* (OUVC 10899) in dorsal view and (b) concave depressions on the internal surface of the nasal bones, indicating olfactory expansions and salt glands.

**FIGURE 21:**
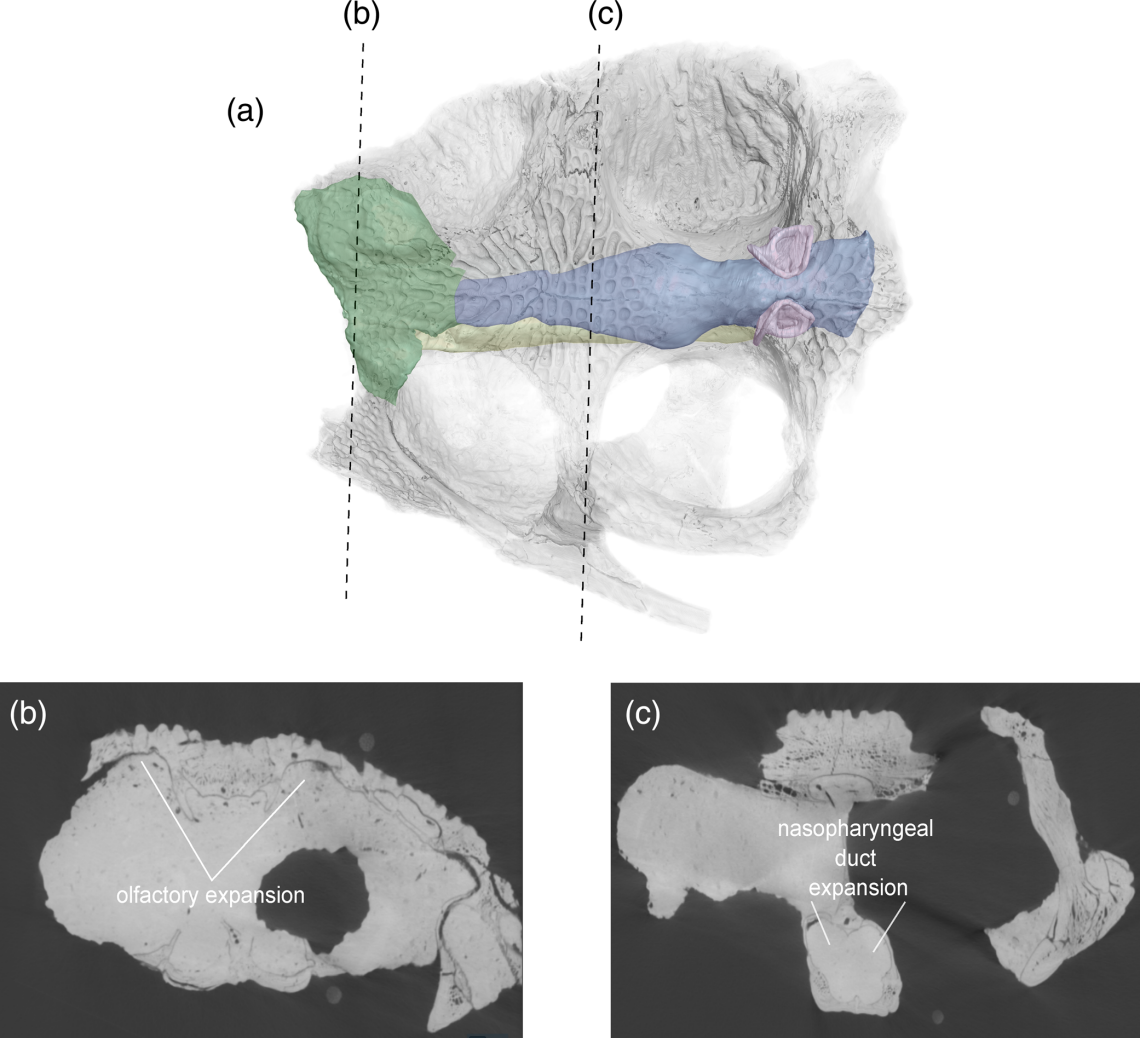
(a) The endocranial anatomy and skull rendering of *Portugalosuchus azenhae* (ML1818) in dorsal view and (b) the concave depressions in the internal surface of the prefrontal and lacrimal bones indicating olfactory expansion and salt glands, and (c) the expansion of the nasopharyngeal duct.

In thalattosuchian crocodyliforms, the paranasal sinus forms concave depressions on the internal surfaces of the prefrontal and lacrimal, and there is a dorsolateral expansion of the olfactory region (Cowgill et al., 2023). Natural endocasts preserve nasal salt glands in this region in metriorhynchid thalattosuchians (Fernández & Gasparini, 2000; Fernández & Herrera, 2021; Herrera et al., 2013), corresponding to the concave depressions of the prefrontal and lacrimal. A significant expansion in this region, medial to the prefrontal, has been interpreted as a result of the enlargement of nasal salt glands in these pelagic crocodyliforms (Cowgill et al., 2023; Pierce et al., 2017). Note that this is different to the large, egg‐shaped structure, referred to as the cartilaginous postconcha, that bulges posterolaterally through the postnasal fenestra in extant crocodylians, located posteriorly to the primary choana, supported by the palatine ventrally, the prefrontal pillar posteriorly and dorsally, and the lacrimals dorsally (see Witmer, 1995; Figures 13, 15, and 17). Therefore, we propose that the concave depressions on the internal surface of the prefrontal and lacrimal, as well as the expansion of the olfactory region medial to the prefrontal (Figure 19c), in *E. lerichei* indicate the possession of salt glands and thus this species was able to tolerate saltwater. This is further supported by the shallow marine geological deposits in which the specimen of *E. lerichei* was recovered (Delfino et al., 2005), as well as the transoceanic distribution of the genus (see below). Extant crocodylians, such as *Crocodylus porosus*, which regularly inhabit saltwater conditions, possess lingual salt glands, but lack nasal salt glands (Taplin et al., 1985). However, the internal surface of the nasals of *Crocodylus porosus* is characterized by concave depressions, which could potentially be osteological correlates for salt glands (Figure 20). Nasal salt glands are present in some extant birds, lizards and marine iguanas, in which they occupy the same region as those reported in Thalattosuchia (Cowgill et al., 2023; Dunson, 1969; fig. 1b) and in *E. lerichei* in this study. Furthermore, we have newly identified these concave depressions in the early Late Cretaceous “thoracosaur” *Portugalosuchus azenhae* (Figure 21).

Today, *Gavialis gangeticus* is only found in freshwater environments (Grigg and Krishner, 2015), and individuals of this species do not possess concave depressions on the internal surfaces of the prefrontal and lacrimal (Figure 19g). However, based on the morphology of the buccal structure of *Gavialis gangeticus*, it has been suggested that this lineage has undergone a secondary reduction from a marine ancestor (Taplin et al., 1985), with saltwater tolerance an apomorphic loss (Delfino et al., 2005). This is consistent with our knowledge of the gavialoid fossil record, with many early diverging members found in marine deposits (Figure 17), such that the adaptation for saltwater tolerance is thought to be plesiomorphic for Gavialoidea (Brochu, 2003; Buffetaut, 1982; Martin et al., 2012).

Further support for marine adaptations arises from the endosseous labyrinth morphology (Figure 7). Schwab et al. (2020) attributed thicker semi‐circular canals in some crocodyliforms to their inhabitation of more pelagic environments, and the semi‐circular canals of *E. lerichei* are thicker than those of *Gavialis* (Figure 7; see Burke & Mannion, 2023). Some pelagic crocodyliforms, such as metriorhynchid thalattosuchians, have equidimensional semi‐circular canals (Schwab et al., 2020). Although the labyrinth of *E. lerichei* does not match the extreme morphology of metriorhynchids (Schwab et al., 2020, 2021), the intermediate morphology might indicate that *E. lerichei* inhabited pelagic environments more frequently than extant species.

The paratympanic sinus reduction observed in *E. lerichei*, and shared by *Tomistoma schlegelii* and some extinct gavialoids, could be controlled by ecological constraints. The pattern of pneumatic reduction shared by these slender longirostrine forms contrasts with that observed in brevirostrine crocodylian forms. In *Alligator mississippiensis*, *Osteolaemus tetraspis*, and *Voay robustus*, the pneumatic recesses are larger and more inflated; the basisphenoid, basioccipital, quadrate and parietal are heavily pneumatized (Dufeau & Witmer, 2015; Perrichon et al., 2023a; Tahara & Larsson, 2022). These differences may reflect several evolutionary tendencies linked to slender longirostry and the aquatic environment. First, that sinus volume and shape seem to be controlled at least partly by skull shape in the crocodylian lineage, leading to the reduction of sinus volume in slender longirostrine forms and associated with narrower braincases, a pattern that has been observed in extant species (Perrichon et al. 2023b). Second, that this loss of pneumatization may result from a gradual change that involved biologic adaptations toward open water environments. Indeed, most known slender longirostrine crocodyliforms show a certain degree of sinus reduction: for instance, dyrosaurid neosuchians occupied freshwater and nearshore environments, and show a significant reduction in dorsal sinus expansion (Erb & Turner, 2021). This tendency becomes extreme in thalattosuchians, where both the shallow water/coastal teleosaurids and the pelagic metriorhynchids completely lack the intertympanic sinus system, possessing no pneumatization in the supraoccipital, parietal, or otoccipital (Brusatte et al., 2016; Herrera et al., 2018; Schwab et al., 2021; Wilberg et al., 2021). Such sinus reduction would influence the buoyancy of the skull, which would likely have an impact on the capabilities of the animal to dive and move rapidly in a 3D underwater environment. This hypothesis could explain a directional selection toward less‐air filled structures in lineages living in marine environments. *Eosuchus* thus possesses a sinus morphology that supports a mostly aquatic lifestyle, possibly restricted to nearshore environments, given that it retains a moderate supraoccipital pneumatisation.

### 5.4 Implications for marine adaptations and transoceanic dispersal in Gavialoidea

As discussed above, morphological evidence for salt‐excretion capability, and therefore for marine adaptation among Gavialoidea, is challenging to retrieve. In extant crocodylians, salt excretion takes place both at the level of the tongue and at the level of the cloaca (e.g., Grigg and Kirschner, 2015), leaving no hard‐tissue evidence for the fossil record. The fossil record of gavialoids showcases several occurrences in nearshore shallow marine deposits (e.g., Jouve et al., 2008; Martin et al., 2014; Salas‐Gismondi et al., 2022; Vélez‐Juarbe et al., 2007) and transoceanic dispersals have previously been hypothesized for several gavialoid lineages. In fact, Vélez‐Juarbe et al. (2007) suggested that early‐diverging gavialoids were probably coastal animals, and that the present‐day restriction of extant species to freshwater environments is a relatively recent adaptation (see also Delfino et al., 2005; Rio & Mannion, 2021). Increasingly, it appears multiple transoceanic dispersals must have taken place among a wide array of gavialoid lineages, explaining the near‐global distribution of the clade, including the presence of sister taxa on distant continents (e.g., Brochu et al., 2004; Delfino et al., 2005; Brochu, 2006; Jouve et al., 2008, 2021; Salas‐Gismondi et al., 2016, 2019; Rio & Mannion, 2021; Groh et al., 2023). Despite the newly proposed phylogenetic topology herein, we fail to identify a clear macroevolutionary pattern of adaptation to the marine environment. Rather, gavialoids recovered from marine deposits are identified within several phylogenetic lineages (Figure 17). This potentially indicates a number of independent acquisitions/losses to marine adaptation, but could also result from the fact that some species might have occupied multiple environments. For example, a species only currently known from non‐marine deposits might also have spent time in oceanic environments, but we are yet to recover fossils of it from marine deposits. Given that most fossil crocodylian species are known from a single locality (e.g., Mannion et al., 2019), such an environmental sampling bias cannot currently be ruled out.

Previous work has already established the need for transoceanic capacity of *Eosuchus*, given its distribution in Europe and North America during the late Paleocene and early Eocene (Taplin and Grigg, 1989; Delfino et al., 2005; Brochu, 2006). During the Paleocene, the Erquelinnes area, from where the specimen of *E. lerichei* was found, was situated on the southern margin of the North Sea Basin, characterized by a shallow, coastal, but fully marine environment (De Coninck et al., 1981; Steurbaut et al., 2003), influenced by nearby deltaic systems (Gibbard & Lewin, 2016). The North Sea Basin shared similarities with its modern configuration, as a partly enclosed basin with several shallow, narrow connections to the Atlantic Ocean; westward through the precursor of the English Channel (Knox et al., 2010; Zacke et al., 2009); and a northward connection via the Viking Graben, Faroe–Shetland Basin and Rockall Trough (Gibbard & Lewin, 2016; Knox et al., 2010). These seaways potentially provided marine dispersal pathways for the *Eosuchus* lineage. During the Thanetian, the connections between the North Sea Basin and the Atlantic Ocean became increasingly restricted, resulting from tectonic uplift and basaltic volcanism caused by the North Atlantic Igneous Province (Jones et al., 2023; Zacke et al., 2009). Following the deposition of the Hannut Formation, the basin eventually became temporarily cut off from the North Atlantic during the Paleocene‐Eocene Thermal Maximum (Jones et al., 2023; Zacke et al., 2009), represented by the overlying Tienen Formation (Steurbaut et al., 2003). This suggests transoceanic dispersal in the *Eosuchus* lineage occurred during the Paleocene, which could have occurred from Europe to North America, or vice versa.

If the presence of a nasal salt gland in *Eosuchus* and *Portugalosuchus* is indeed supported by our observations, this provides the first anatomical support for the capacity of extinct gavialoids to live in and disperse via the marine environment. Given that the earliest diverging and stratigraphically oldest gavialoids either have evidence for a nasal salt gland and/or have been recovered from marine deposits (Figure 17), this suggests that the capacity for salt excretion might be ancestral for Gavialoidea. The geographical distribution of the late Oligocene–Miocene North American + European clade *Thecachampsa* + *Gavialosuchus*, as well as the appearance of gryposuchines and related taxa in the late Paleogene–early Neogene of Central and South America, suggests that at least some gavialoids maintained this adaptation. But other instances of gavialoids in freshwater environments in the Neogene (e.g., *Penghusuchus*) could indicate that this capacity had been lost or reduced. Our topology currently indicates that there might therefore have been more than one independent loss/reduction in the capacity for salt excretion and marine adaptation in gavialoids, although this must remain tentative until a phylogenetic hypothesis exists that is stratigraphically congruent with molecular divergence time estimates.

## 6 CONCLUSIONS

We present the first evaluation of endocranial anatomy of the late Paleocene northwestern European gavialoid crocodylian species *E. lerichei*. Based on CT‐scan data, we show that this species potentially possessed salt glands, based on concave depressions in the prefrontal and lacrimal. This would provide the first anatomical evidence for the capacity of extinct gavialoids to live in and disperse via the marine environment, supporting previous hypotheses of transoceanic dispersal based on geographical distributions and environments of deposition. Incorporation of internal anatomical information, as well as new interpretations of external anatomy, enable a revised phylogenetic placement for *Eosuchus* within Gavialoidea. This suggests that it was not part of the “thoracosaur” group, for which we find renewed support for the monophyly of thoracosaurs.

## AUTHOR CONTRIBUTIONS

**Paul M. J. Burke:** Methodology; investigation; writing – original draft; project administration; data curation; formal analysis; writing – review and editing; visualization; software. **Sophie A. Boerman:** Methodology; investigation; data curation; writing – review and editing; formal analysis; funding acquisition. **Gwendal Perrichon:** Writing – review and editing; methodology; software; data curation; investigation; formal analysis; visualization. **Jeremy E. Martin:** Supervision; writing – review and editing. **Thierry Smith:** Supervision; writing – review and editing. **Johan Vellekoop:** Supervision; writing – review and editing. **Philip D. Mannion:** Supervision; writing – review and editing; funding acquisition; conceptualization.

## Supporting information


**DATA S1:** Supporting Information.

**DATA S2:** Supporting Information.
