# Supplementary material for: Endocranial anatomy and phylogenetic position of the crocodylian Eosuchus lerichei from the late Paleocene of northwestern Europe and potential adaptations for transoceanic dispersal in gavialoids
Source: Anat Rec (Hoboken). 2024 Sep 3;308(2):636–70. doi: 10.1002/ar.25569 (PMC11725715; doi:10.1002/ar.25569)
Supplement: Supplementary file 1 — DATA S1: Supporting Information. [file AR-308-636-s002.docx]

Supplementary Material for “Endocranial anatomy and the phylogenetic position of the gavialoid crocodylian *Eosuchus lerichei* from the late Paleocene of Belgium and potential adaptations for oceanic dispersal in gavialoids”

**TABLE 1**: Measurements of the cranial endocasts and labyrinths of crocodyliform taxa.

| **Measurements (mm)** | ***Eosuchus lerichei***  (IRSNB R49)  This study | ***Gavialis gangeticus***  (FLMNH UF 118998)  Pierce *et al*. (2017) | ***Tomistoma schlegelii***  (TMM M6342)  Burke & Mannion (2023) |
| --- | --- | --- | --- |
| Skull width at cerebrum (b/w postorbitals) | 95 | 168 | 68 |
| Cephalic flexure angle | 167 | 150 | 134 |
| Pontine flexure angle | 170 | 154 | 134 |
| Brain endocast length | 115 | 146 | 97 |
| Olfactory tract length | 52 | 55 | 47 |
| Cerebrum width | 26 | 32 | 27 |
| Pituitary width | ? | 6 | 5 |
| Pituitary height | ? | 9 | 8 |
| Pituitary length | ? | 11 | 14 |
| Maximum width of labyrinth | 17 | 21 | 18 |
| Maximum height of labyrinth | 17 | 21 | 17 |
| Endosseous cochlea length | ? | 9 | 10 |
| Anterior semi-circular canal area | 43 | 36 | 17 |
| Posterior semi-circular canal area | 9 | 15 | 5 |
| Lateral semi-circular canal area | 12 | 22 | 4 |

**TABLE 2:** Neurovascular canal measurements

| **Neurovascular canals measurements (mm)** | ***Eosuchus lerichei***  (IRSNB R49)  This study | ***Tomistoma schlegelii***  (TMM M6342) | ***Gavialis gangeticus***  (FLMNH UF 118998) |
| --- | --- | --- | --- |
| Trigeminal foramen diameter | 4.6-3.2 | 6 | 5.7 |
| Trigeminal fossa length/width | 8.8/6.1 | 9/5.6 | 8/9 |
| Tympanic branch of CNV, diameter | 1.5 | 1.1 | 1 |
| Tympanic branch of CNV, length | 9 | 6.7 | *Not reconstructed* |
| Supraorbital branch of CNV, diameter | 0.65 | *Not reconstructed* | *Not reconstructed* |
| Ophtalmic branch of CNV, diameter | 2.1-4.1 | *Not reconstructed* | *Not reconstructed* |
| Mandibular branch of CNV, diameter | 1-2.8 | *Not reconstructed* | *Not reconstructed* |
| Abducens nerve, diameter | 0.7 | 0.8 | 0.8 |
| Abducens nerve, length | 5.6 | 9.3 | 13.5 |
| Facial nerve foramen, diameter | 0.9 | 1.6 | 1.4 |
| Facial nerve, diameter | 1.6 | 0.9 | 1.4 |
| Palatine branch of the facial nerve, diameter | 0.8 | 0.7 | 1.7 |
| Posterior foramen of CN IX-XI, diameter | 3.5 | 4.2 | 5.8 |
| Sympathetic nerve, diameter | 1.8 | 1.3 | 2.8 |
| Glossopharyngeal nerve, diameter | 0.5 | 1.2 | 0.7 |
| Vagus and spinal accessory nerves, diameter | 1 | 1.2 | 2 |
| Anterior hypoglossal nerve, diameter | 0.5 | 1 | *Not reconstructed* |
| Posterior hypoglossal nerve, diameter | 1.5 | 1.7 | 1.6 |
| Cranioquadrate passage, diameter | 4.8 | *Not reconstructed* | *Not reconstructed* |
| Cerebral carotid arteries, diameter | 3 | 3 | 2.3 |
| Arterial branch linking the carotid and the stapedial artery, diameter | 1.6 | *Not reconstructed* | 1.3 |

# **Changes made to the matrix of Burke et al. (2024)**

**Character scoring for *Eosuchus lerichei***

Characters that differ from the scoring in Burke et al. (in review) are indicated in bold.

0.653 **1.071** **0.769** **0.538** **0.400** **0.214** **0.588** **0.694** 0.000 **0.574** **0.846 0.233** 0.410 1.733 3.646 **1.632** 16.000-17.000 ? ? ? ? ? ? ? ? ?

00000 ??000 00??1 **?**0001 **2**001**0** 000?0 100**1**0 0010**1** **0**0**0**00 010**2**0 **?**?**0**00

0010**1** **?1**00? 10010 0**110**? **000**0**0** 1011**1** 000**10** 00201 00000 10001 00000 00000

**2**0**1**03 70112 00000 0**0**001 **0**00**0**0 **1**00?0 10000 0000**1** 000**0**1 12**0**00 0**00**00 0**0**00**0**

**0**?**?**?**0** **0**?0?? **1???0** 103**03** 0?21? 0**?**?**?**? 00**???** **?????** ??**???** **?????** **???**?**?** **?**????

????? ???1? ?**??1**1 **1**0**0**?1 ???**?**? ????? **?**???? ??**?**?**?** **???**11 **?**???? ????? ?????

??**?1**1 **1**???**0**

## **Coding correction of character 86 (Parietal, recess communicating with pneumatic system: present (0); absent (1)).**

Extant species, based on Perrichon et al. (2023a).

*Alligator*, all species: 0

*Caiman*, all species: 0

*Melanosuchus*: 0

*Paleosuchus*, all species: 0

*Crocodylus niloticus*: variable [01]

*Crocodylus rhombifer*: variable [01]

*Crocodylus acutus*: variable [01]

*Crocodylus novaeguineae*: variable [01]

*Crocodylus porosus*: variable [01]

*Crocodylus siamensis*: 0

*Crocodylus palustris*: 0

*Osteolaemus tetraspis*: 0

*Mecistops cataphractus*: 0

*Gavialis gangeticus*: 0

*Tomistoma schlegelii*: 1

Other extant species are coded ? in absence of close examination of CT-scans of adult specimens.

Fossil species:

*Eosuchus lerichei*: 1

*Gryposuchus neogaeus*: 1 (Bona et al., 2017)

*Portugalosuchus azenhae*: 1 (Puértolas-Pascual et al., 2023)

*Agaresuchus*: ? (Serrano-Martinez et al., 2021; Puértolas-Pascual et al., 2022)

*Arenysuchus*: ? (Puértolas-Pascual et al., 2022)

*Lohuecosuchus megadontos*: 0 (Serrano-Martinez et al., 2018)

*Diplocynodon tormis*: 0 (Serrano-Martinez et al., 2019)

*Voay robustus*: 0 (Perrichon et al., 2023b)

# **References**

Bona, P., Paulina-Carabajal, A., & Gasparini, Z. (2015). Neuroanatomy of *Gryposuchus neogaeus* (Crocodylia, Gavialoidea): a first integral description of the braincase and endocranial morphological variation in extinct and extant gavialoids. *Earth and Environmental Science Transactions of the Royal Society of Edinburgh*, 106 (4), 235-246. <https://doi.org/10.1017/S1755691016000189>

Burke, P.M.J., Nicholl, C.S.C., Pittard, B.E., Sallam, H., & Mannion, P.D. (2024) The anatomy and taxonomy of the North African Early Miocene gavialoid ‘*Tomistoma*’ *dowsoni* and the phylogenetic relationships of gavialoids (In press)

Perrichon, G., Hautier, L., Pochat-Cottilloux, Y., Raselli, I., Salaviale, C., Dailh, B., Rinder, N., Fernandez, V., Adrien, J., Lachambre, J. & Martin, J.E. (2023) Ontogenetic variability of the intertympanic sinus distinguishes lineages within Crocodylia. *Journal of Anatomy*, 242 (6), 1096–1123. <https://doi.org/10.1111/joa.13830>

Puértolas‐Pascual, E., Kuzmin, I. T., Serrano‐Martínez, A., & Mateus, O. (2023). Neuroanatomy of the crocodylomorph *Portugalosuchus azenhae* from the late cretaceous of Portugal. *Journal of Anatomy*, 242(6), 1146–1171. <https://doi.org/10.1111/joa.13836>

Puértolas-Pascual, E., Serrano-Martínez, A., Pérez-Pueyo, M., Bádenas, B., & Canudo, J. I. (2022). New data on the neuroanatomy of basal eusuchian crocodylomorphs (Allodaposuchidae) from the Upper Cretaceous of Spain. *Cretaceous Research*, 135, 105170. <https://doi.org/10.1016/j.cretres.2022.105170>

Serrano-Martínez, A., Knoll, F., Narváez, I., Lautenschlager, S., & Ortega, F. (2018). Inner skull cavities of the basal eusuchian *Lohuecosuchus megadontos* (Upper Cretaceous, Spain) and neurosensorial implications. *Cretaceous Research*, 93, 66-77. <https://doi.org/10.1016/j.cretres.2018.08.016>.

Serrano-Martínez, A., Knoll, F., Narváez, I., & Ortega, F. (2019). Brain and pneumatic cavities of the braincase of the basal alligatoroid *Diplocynodon tormis* (Eocene, Spain). *Journal of Vertebrate Paleontology*, 39 (1), e1572612. <https://doi.org/10.1080/02724634.2019.1572612>.

Serrano-Martínez, A., Knoll, F., Narváez, I., Lautenschlager, S. & Ortega, F. (2021). Neuroanatomical and neurosensorial analysis of the Late Cretaceous basal eusuchian *Agaresuchus fontisensis* (Cuenca, Spain). *Papers in Palaeontology*, 7, 641–656
